# Sampling Strategies and Biodiversity of Influenza A Subtypes in Wild Birds

**DOI:** 10.1371/journal.pone.0090826

**Published:** 2014-03-05

**Authors:** Sarah H. Olson, Jane Parmley, Catherine Soos, Martin Gilbert, Neus Latorre-Margalef, Jeffrey S. Hall, Phillip M. Hansbro, Frederick Leighton, Vincent Munster, Damien Joly

**Affiliations:** 1 Wildlife Conservation Society, Bronx, New York, United States of America; 2 Canadian Cooperative Wildlife Health Centre – Department of Pathobiology, University of Guelph, Guelph, Ontario, Canada; 3 Environment Canada, Science & Technology Branch, Saskatoon, Saskatchewan, Canada; 4 Centre for Ecology and Evolution in Microbial Model Systems (EEMiS), Linnaeus University, Kalmar, Sweden; 5 Department of Population Health, College of Veterinary Medicine, Southeastern Cooperative Wildlife Disease Study, University of Georgia, Athens, Georgia, United States of America; 6 United States Geological Survey (USGS) National Wildlife Health Center, Madison, Wisconsin, United States of America; 7 Priority Research Centre for Asthma and Respiratory Disease, University of Newcastle and Hunter Medical Research Institute, Newcastle, New South Wales, Australia; 8 Canadian Cooperative Wildlife Health Centre, Western College of Veterinary Medicine, University of Saskatchewan, Saskatoon, Saskatchewan, Canada; 9 Laboratory of Virology, Division of Intramural Research, National Institute of Allergy and Infectious Diseases, National Institutes of Health, Hamilton, Montana, United States of America; 10 Metabiota, Nanaimo, British Columbia, Canada; INIAV, I.P.- National Institute of Agriculture and Veterinary Research, Portugal

## Abstract

Wild aquatic birds are recognized as the natural reservoir of avian influenza A viruses (AIV), but across high and low pathogenic AIV strains, scientists have yet to rigorously identify most competent hosts for the various subtypes. We examined 11,870 GenBank records to provide a baseline inventory and insight into patterns of global AIV subtype diversity and richness. Further, we conducted an extensive literature review and communicated directly with scientists to accumulate data from 50 non-overlapping studies and over 250,000 birds to assess the status of historic sampling effort. We then built virus subtype sample-based accumulation curves to better estimate sample size targets that capture a specific percentage of virus subtype richness at seven sampling locations. Our study identifies a sampling methodology that will detect an estimated 75% of circulating virus subtypes from a targeted bird population and outlines future surveillance and research priorities that are needed to explore the influence of host and virus biodiversity on emergence and transmission.

## Introduction

The recent emergence of zoonotic avian influenza H7N9 in China [1] and H5N8 in South Korea [2] has highlighted the need to understand the genetic and phenotypic diversity of avian influenza viruses (AIVs), including those of wild bird reservoirs, beyond the traditional focus on highly pathogenic avian influenza (HPAI) strains. Influenza A virus is a major pathogen from a public, veterinary, and wildlife health perspective, yet there are no comprehensive reviews of AIV subtype diversity in birds and the evolutionary drivers of virus diversity are not well understood. Diversity exists within each of the eight genetic segments of the influenza A genome and between the different combinations of those segments that arise from reassortment events. Our focus is on the different combinations of the hemagglutinin (HA) and neuraminidase (NA) genetic segments (hereafter referred to as subtype diversity), and the number of unique HA and NA combinations (hereafter referred to as subtype richness), derived from available surveillance data. An inventory of subtype diversity and richness is a first step towards understanding what drives virus richness, which may help predict emergence events [3], [4].

Species accumulation curves are powerful tools in ecology [5], but have only recently been introduced for use in pathogen research, with one study of virus diversity [6]. Ecologists use accumulation curves to compare species richness among locations when sampling efforts are dissimilar, which is the norm for global AIV surveillance in wild birds [7]. With 16 known HA subtypes and nine NA subtypes recorded in wild birds, there are 144 possible HA/NA combinations. The suite of 144 possible AIV subtypes makes AIV an excellent candidate to evaluate the utility and limitations of sample-based accumulation curves towards estimating plateaus of virus richness and sampling targets. We employed these curves, acknowledging their limitations, to examine reported differences between subtype richness for large surveillance collections (arbitrarily defined as those with at least 5,000 birds and five sampling periods).

The purpose of this research is to describe the current global diversity of AIV subtypes, explore patterns of virus subtype richness, and probe the benefits and limitations of sample-based accumulation curves to study AIV subtype richness in general.

## Methods

### a. GenBank

On 25 September 2012, we downloaded 11,870 distinct avian records with subtype data (HA and NA gene segments) from the Influenza Virus Database (http://www.ncbi.nlm.nih.gov/genomes/FLU/Database/nph-select.cgi?go=database) in GenBank [8], date collected from 1902 through 2012. Records were classified into wild (including migratory birds), domestic (including poultry and farmed), feral, captive (including birds in trade, in zoos, and pets) or unknown, based on a variety of resources including GenBank records, GenBank linked publications, sampling location, and species distributions. Flyways were defined according to the North American Flyway Directory [9] and a country-based division into North America (USA and Canada only), Latin America, Europe, Africa, Asia, and Australasia. Maps were created in ArcGIS 9.3 (ESRI, Inc.) and Photofiltre 6.5.1.

We used the GenBank data to examine the relationship between the detection of a particular AIV subtype from domestic birds and host range of that subtype in wild birds (host genus richness). We used a generalized linear model with a binomial distribution and a logit link function to calculate the odds ratio of isolating a particular subtype in domestic birds (presence in domestic birds) based on observed wild bird host genus richness. We adjusted for effort by including the number of GenBank records for each subtype (effort). Statistical analysis and figures were completed in R version 2.15.3 [10]. Thus, for each HA/NA subtype we modeled:

f(presence in domestic birds)  =  α + β_1_ (wild bird host genus richness) + β_2_ (effort).

### b. Modeling subtype richness

We collected published and non-published surveillance efforts that non-discriminately tested for AIV subtype. Many surveillance programs and studies did not meet the criteria, such as those that screened samples by real-time reverse-transcriptase PCR (RT-PCR) and only subtyped those samples that tested positive for specific subtypes (e.g. H5 or H7). Full descriptive information (including associated references, location, prevalence, detected AIV richness, sampling and testing methodology, number of sampling periods, and sample years) for each of these studies can be found in the electronic supplementary material (Supplementary Tables S1 and S2). Location, year of sampling, and authorship were tracked to avoid duplicate reporting, resulting in non-overlapping studies from the Northern Hemisphere (n = 41) and the Southern Hemisphere (n = 9). These studies relied on virus isolation or RT-PCR methods for AIV detection in cloacal, fecal, or tracheal samples. Virus isolates were further characterized by HA or NA inhibition assays, subtype specific RT-PCR, or by sequencing the HA and NA gene segments of the virus isolate. Viruses partially subtyped (those with only HA or NA subtype) were not included in the analysis.

#### i. Sample-based accumulation curves

Studies with at least five sampling periods and 5,000 birds tested overall were identified and nonparametric AIV subtype richness was predicted for each study (Table1). The baseline effort measure (5,000 birds) was established to focus on studies with the largest comparable sample sizes. We restricted the sampling periods to maintain consistency of effort among studies in addition to sample size, and to limit the analysis to a manageable number of major studies. We also predicted cumulative unique subtype richness from GenBank by year from 1959 to 2012.

**Table 1:** Overview of seven studies with at least 5,000 birds collected over at least five sampling events.

Location (author, year)	AIV richness/ sample size (per 1000)	Analysis method	Sampling period	Bird families (positive/total, % positive)
**Portugal** [51]	20/5691 (3.5)	Cloacal & oropharyngeal + mRT-PCR + isolation + sequencing	2005–2009 (year-round)	Total (93/5691, 1.63%)
**Mongolia** 1	28/5831 (3.9)	Fecal sample + isolation + HI & NI test + RT-PCR + sequencing	July 2009–October 2012 (May, Jun, Jul, Aug, Sep, Oct)	Anatidae (80/5731, 1.4%); Laridae (0/100, 0%)
**Egypt** 2 [50]	17/6070 (2.8)	Cloacal swab + RT-PCR + isolation + HI & NI test + sequencing	2003–2007 (Sep–Feb)	Anatidae majority (9.4%)
**Canada**, Alberta [34]	44/9195 (4.8)	Cloacal^3^ + isolation + HI & NI test	1976–1983 (Aug)	Anatidae majority (2275/9195, 24%)
**Sweden** [14]	74/18645 (4.0)	Cloacal swab + real-time RT-PCR + isolation + HI & NI test + sequencing	2002–2009 (Mar–Dec)	*Anas platyrhynchos* (2463/18645, 13.2%)
**Europe** 4	29/24516 (1.2)	Cloacal swab + real-time RT-PCR + isolation + HI & NI test	1998–2005 (year-round)	Total (612/24516, 2.5%); *Anas platyrhynchos* (325/4398, 7.4%)
**Taiwan** 5 [37]	46/44786 (1.0)	Fecal sample + isolation + HI & NI test + RT-PCR + sequencing	1998–2011 (year-round)	Anatidae (229/20812, 1.1%); Shorebirds (3/6435, 0.05%); Laridae (2/617, 0.32%); Ardeidae (2/825, 0.24%); Other birds (1/598, 0.17%)

1 Unpublished data provided by Martin Gilbert 10 February 2013.

2 Richness estimated based on data thru 2007, population prevalence based on data through 2009.

3 Sampling method was not reported but based on historical sampling patterns suggest it was cloacal.

4 Provided by Vincent Munster 21 December 2012. These data excluded Ottenby Mallard data reported under Sweden.

5 Extended data provided by Meng-Chu Cheng 12 November 2012. Bird families based on published data from Cheng et al. 2010.

Positive samples used to calculate prevalence (positive/total) may not all have been fully subtyped.

We used EstimateS v 8.2.0 [11] to generate a presence-absence accumulation function of subtypes and calculated the nonparametric estimate of subtype richness with 95% confidence intervals using the Chao2 estimate and 50 randomizations with replacement [6]. Bias-corrected Chao2 was calculated unless the coefficient of variation for the incidence distribution was less than 0.5, in which case Classic Chao2 was calculated. We applied Chao's nonparametric estimator of sufficient sampling to calculate the minimum number of birds necessary to detect 75% of the estimated asymptotic subtype richness [12]. A 75% target was selected because reaching the asymptote is problematic [13]. Datasets are staged on the Knowledge Network for Biocomplexity repository (http://doi.org/10.5063/F1HT2M7Q).

#### ii. Drivers of AIV richness

We examined the attributes of the 50 studies and identified variables associated with richness that could be reliably extracted and analyzed as covariates. Our attempts to isolate measures of host diversity (percentages of Anseriformes and Charadriiformes) were hampered by data availability reducing the number of studies to 41, but we were able to extract AIV prevalence and duration of study (years). We used linear mixed models (R library lme4, function lmer) to examine the effects of interactions and to estimate the variance of subtype richness associated with region and selected the best model based on Bayesian Information Criterion (Supplementary Table 3).

### c. Sampling methods and ethic statements for data provided by co-authors

Samples from Sweden were collected from wild ducks at an important stopover site in the island of Öland (56°12′N 16°24′E) located in the Northwest European flyway [14]. Breeding grounds of the duck populations using the site are Baltic countries and Northwestern Russia [15]. Ducks were caught using a live-duck trap and all handling of birds was performed by trained ornithologists from Ottenby Bird Observatory. Samples were collected in transport media [16] and kept frozen at −70°C until analysis [17]. The sampling protocol was approved by Linköping Animal Research Ethics Board (permit numbers 8–06, 34–06, 80–07, 111–11, 112–11) in accordance with national legislation.

In the European Union, expert ornithologists trapped birds using duck decoys, duck traps, wader funnel traps, mist nets, clap nets, cannon nets, or Helgoland traps. The majority of samples were obtained from migratory birds during fall migration at long-term sampling sites in the Netherlands [18]. Cloacal swabs were collected using sterile cotton swabs and stored in transport medium [16] and shipped to the laboratory where they were stored at −80°C for analysis. The handling of birds within the European Union study was in accordance with national and international guidelines that were approved by an independent Animal Ethics Committee of the Erasmus Medical Center (Stichting DEC Consult) under permit number 122-09-20.

Mongolian sample collections focused on environmental fecal samples, negating the need for national permits for the capture and handling of wild birds. Sampling took place on state-owned land in 27 locations in the East region of Mongolia, four in the North-Central region and three in the West region, based on the nomenclature used in Gilbert et al. 2012 [19]. The project was approved by the University of Minnesota, Institutional Animal Care and Use Committee (Protocol 1006A84613). Work was completed under the authorization of the Mongolian State Central Veterinary Laboratory.

## Results

### a. Subtype diversity and richness reported in GenBank

In the 1990s the number of GenBank submissions for AIV viruses with fully sequenced HA and NA genes began to increase. It peaked in 2007, and since that time there has been a steep drop in the number of wild bird sequences deposited and a similar but less sustained drop in the number of poultry sequences deposited (Supplementary Figure S1). A total of 117 HA/NA subtype combinations have been recorded in the Influenza Virus Database for all birds, wild and domestic.

#### i. Subtypes unique to bird orders, families, and genera

In 4,163 wild bird AIV sequences, 112 subtypes were identified (Supplementary Table S4), of which 49 (44%) were also found in domestic birds. Five subtypes not observed in wild birds were H6N7 from a domestic goose, H9N8 from a chicken and unknown duck species, and H8N2, H8N7, and H15N8 found in unknown ducks (see Supplementary Table S5 for a list of specific subtypes within each bird order).

The highest richness of subtypes from wild birds came from the order Anseriformes (n = 101) including 33 subtypes that were not submitted from any other bird order. All of these subtypes were found in wild birds of the family Anatidae and eight were shared with domestic birds (Supplementary Table S6). Charadriiformes had 70 subtypes, 10 of which were unique to this order in wild birds (Supplementary Table S7). One subtype, H15N6, was only found in Procellariiformes, sequenced from a shearwater sp. collected in Australia in 1979. Unique AIV subtypes were not found from any other order of wild birds (Supplementary Table S5). The remaining 68 subtypes were isolated from more than one order (61% of the 112 subtypes). Of these, all subtypes were found in Anseriformes (primarily from birds of the family Anatidae (*Anas* spp.)), 60 were also found in Charadriiformes, and 1–7 were found in other bird orders (Supplementary Table S5).

Based on those submissions for which species information was available, the top five wild host species for subtype richness (n) were: Mallard - *Anas platyrhynchos* (89), Ruddy Turnstone - *Arenaria interpres* (45), Northern Pintail - *Anas acuta* (43), Northern Shoveler - *Anas clypeata* (35), and Blue-winged Teal - *Anas discors* (33).

#### ii. Subtypes found in wild and domestic birds

**Figure 1:**
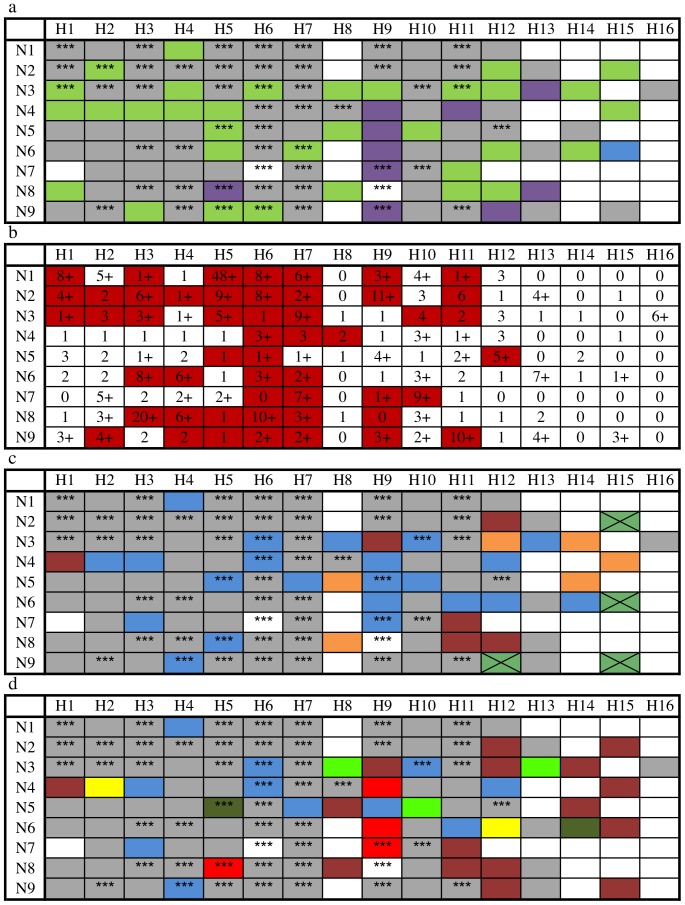
GenBank data on subtypes displayed with host and geographic information. (*a*) Subtypes found in Anseriformes (green), Charadriiformes (purple), Procellariiformes (blue), more than one order (gray), and occurrence in domestic birds (***); (*b*) richness of identifiable wild bird genera (of 81 examined) associated with each subtype (+ indicates found in at least one family where genus was not determinable) and occurrence in domestic birds (red); (c) distribution of subtypes unique to the continents of North America (blue), Europe (brown), Asia (tan), Australasia (green with black X), and across multiple continents (gray); and (d) distribution of subtypes unique to the North American Pacific Flyway (light green), Central Flyway (yellow), Mississippi Flyway (dark green), and Atlantic Flyway (red). Also indicated are subtypes unique to North America but found in multiple flyways (blue), subtypes unique to other continents (brown), and subtypes found across multiple continents (gray).

Of the 49 subtypes found in both wild and domestic birds, 46 (94%) were found in wild Anseriformes, and 37 (76%) were isolated from wild Charadriiformes (Figure 1a). Host genus richness by subtype for 3,628 records was compiled (Figure 1b). GenBank data was not conclusive enough to definitively identify the taxonomic host genus on 13% of the wild bird records. The model minimized differences between the observed and expected occurrence of a subtype in domestic birds under the Hosmer-Lemeshow goodness-of-fit test (p = 0.586) [20]. After adjusting for sampling effort for each subtype, we estimate that for every additional wild bird genus from which a subtype was isolated (i.e. host genus richness), the odds of finding that subtype in domestic birds increased 70% (odds ratio [95% CI]  = 1.70[1.38, 2.10]). Effort was not significant with odds ratio 1.00[0.99,1.01]. In other words, the more frequently a subtype is found in different wild host genera the more likely it will be found in domestic birds.

#### iii. Subtypes unique to continents and flyways

From birds sampled since 1959, scientists have deposited 2,587 (94 subtypes) wild bird AIV sequences, with identified subtypes in the Influenza Virus Database, from North American flyways, 565 from Europe (67 subtypes), 856 from Asia (61 subtypes), 61 from Africa (16 subtypes), 68 from Australia and New Zealand (21 subtypes), and 26 from Latin America (14 subtypes). Twenty-two subtypes were unique to North America, with nine found only in Anseriformes and six found only in Charadriiformes (Figure 1c and Supplementary Table S8). Six subtypes were unique to Europe, all found only in Anseriformes. Asia had six unique subtypes, five found only in Anseriformes and one found only in Charadriiformes. Australia and New Zealand had four unique subtypes, one each found in Anseriformes, Charadriiformes, and Procellariiformes, and one found in all three orders. No subtypes were unique to Latin America or Africa, and 74 were found on multiple continents.

Within North America, 802 (61 subtypes) wild bird AIV sequences were reported from the Pacific, 439 (52 subtypes) from the Central, 660 (54 subtypes) from the Mississippi, and 686 (65 subtypes) from the Atlantic flyways. Specific subtypes unique to each are shown in Figure 1d (see Supplementary Table S9 for overview by bird order).

### b. Measuring effort and estimating richness

#### i. Global richness during 1977-2012 in wild birds

**Figure 2:**
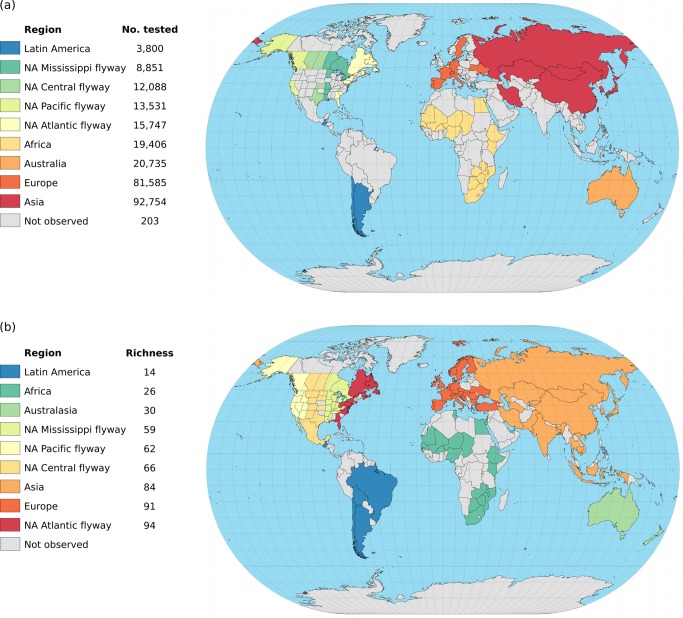
Maps of global sampling effort and observed richness for nine regions. (*a*) Global sampling effort (total number of birds tested) by region reported in 50 studies that non-discriminately tested for AIV subtype. If we did not identify studies that met the inclusion criteria for a country, state (USA only), or province (Canada only) we report it as not observed. (*b*) Total richness (number of AIV subtypes detected) by region based on GenBank records and 50 studies. A country, state (USA only), or province (Canada only) is not observed if we did not identify studies that met the inclusion criteria and if no subtypes were reported in GenBank.

We incorporated 50 studies that totaled 268,700 wild birds sampled around the world between 1977 and 2012 (Figure 2a). Our survey identified 116 subtypes of which 102 were represented among the wild bird sequences extracted from GenBank and 14 were not (H1N7, H6N7, H8N1, H8N2, H8N6, H8N7, H9N8, H12N7, H13N1, H13N4, H13N7, H14N7, H15N1, and H16N8). When unique subtypes from the 50 studies were combined with the GenBank submissions, the greatest subtype richness was in North America (Atlantic Flyway), Europe, and Asia (Figure 2b).

#### ii. Large collections (>5000 birds) and sampling targets

We identified seven distinct studies, all from the Northern Hemisphere, which sampled at least 5,000 birds during at least five sampling periods and indiscriminately tested for all AIV subtypes. A brief description of these studies that predominately sampled Anatidae, is provided (Table1). Egypt had the lowest richness of AIV subtypes at 17, followed by Portugal at 20. Sweden had the highest detected richness for one location (n = 74) [14].

**Figure 3:**
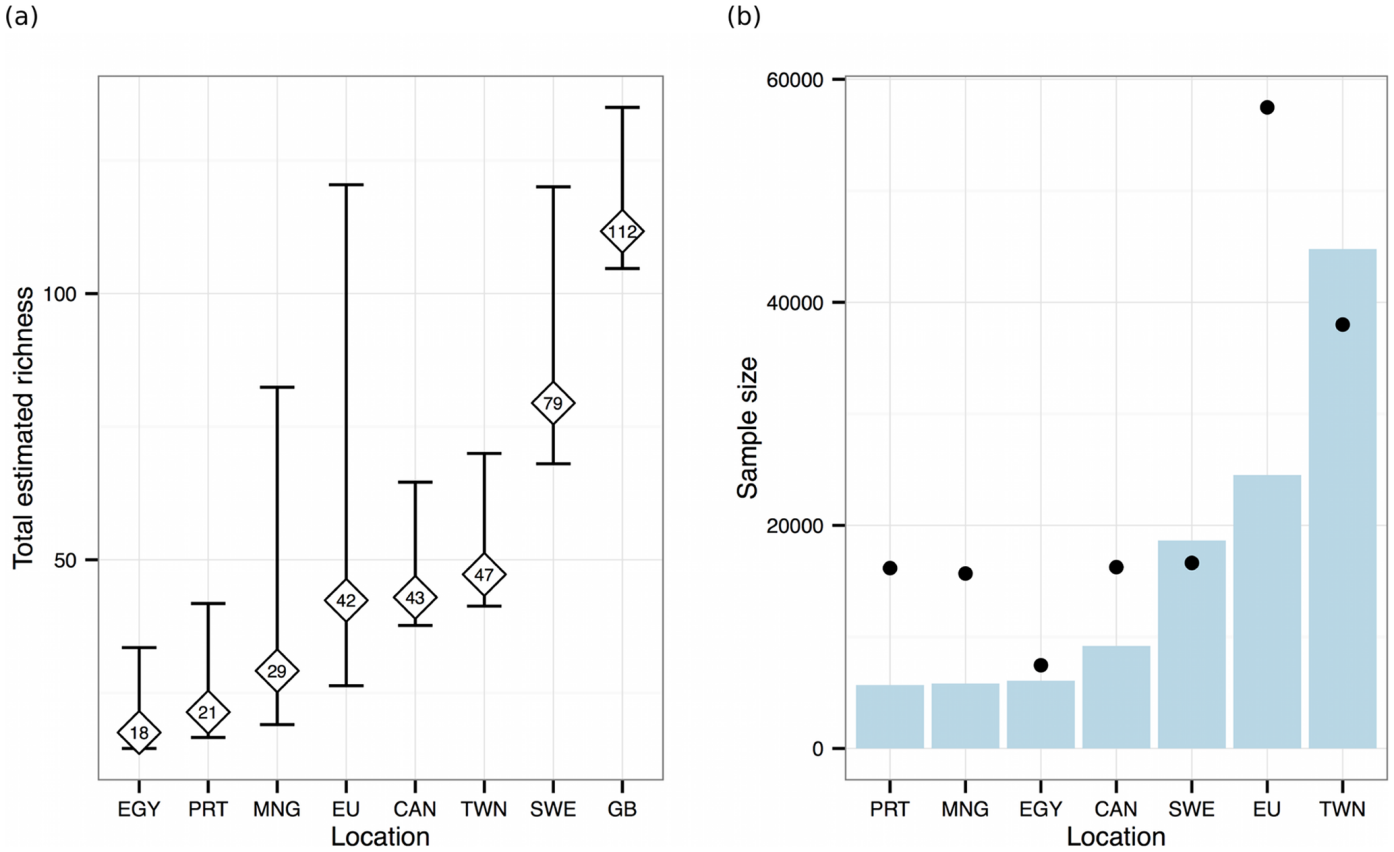
Subtype richness and sampling effort varies among studies. (a) Predicted AIV subtype richness (diamonds) with 95% confidence intervals based on the mean Chao2 richness estimator for seven distinct study locations for which the criteria of five sampling events and >5,000 birds tested non-discriminately for AIV subtypes were met (Table? 1). Further, predicted AIV subtype Chao2 richness estimates are displayed from GenBank. (b) The sample size at each study location (blue bar) and minimum sufficient sampling size necessary to capture 75% of total estimated AIV richness (black dot). EGY  =  Egypt [50], PRT  =  Portugal [51], MNG  =  Mongolia (this study), EU  =  European Union (this study), CAN  =  Alberta, Canada [34], SWE  =  Sweden [14], TWN  =  Taiwan [37], and GB  =  sequences with subtypes deposited in GenBank (1959–2012).

The nonparametric estimates of 95% confidence intervals for total predicted AIV subtype richness were not overlapping for many sites (Figure 3a). Predicted subtype richness, based on rarefaction alone, was significantly different for some paired comparisons among Egypt, Portugal, Canada, Taiwan, and Sweden. The non-parametric mean Chao2 richness estimator [95% CI] of AIV subtypes was 112[104, 135] based on occurrence of GenBank sequences by year.

The sufficient sampling analysis showed that in five out of the seven studies sampling targets set to acquire 75% of total AIV subtype richness (75% target) were below 20,000 birds (Figure 3b). Taiwan (n = 38,008) and the European Union (n = 57,480) required the highest numbers of birds to reach the 75% target whereas Egypt required the fewest birds (n = 7,464). Under their respective sampling methodologies, among the seven studies the 75% target was only achieved for the studies in Sweden and Taiwan. The relationship between the 75% target sample size and total predicted richness showed that some studies and locations were more efficient at detecting richness (Supplemental Figure S2).

#### iii. Examination of effort and other drivers associated with richness

Increasing the number of birds sampled, duration of study (years), and prevalence of AIV were associated with increased AIV richness (Supplementary Table S3). Percentages of Anseriformes and Charadriiformes sampled did not improve the model fit. ANOVA analysis suggests that 28% of richness variance can be explained by geographic region (North America, Latin America, Europe, Asia, Australia, and Africa). Best model fit was achieved with a three-way interaction term for number of birds sampled, duration of study, and AIV prevalence. Increasing each interaction term was associated with greater AIV richness.

## Discussion

### a. GenBank database

#### i. Observations and findings

GenBank is an important data source with characteristics and biases that reflect human health concerns and funding streams. Up until 1995, there was a steady low level of AIV sequences from wild bird hosts being deposited into GenBank. Then the number of deposits substantially increased with domestic bird sequences and specifically H5N1 subtype sequences predominating. After 2007, the number of both wild and domestic submissions declined [21].

The GenBank data demonstrate that the majority of wild bird subtypes were not specific to an avian order or continent; 61% (68/112) of wild bird subtypes were identified in >1 order and 66% (74/112) were identified on >1 continent. Globally, 79% (89/112) of wild bird subtypes were found in Mallards. However, our results show that increasing host genus range was significantly associated with increases in the odds of finding a subtype in domestic birds. Although this may reflect some unadjusted bias in the database associated with the high propensity to sequence wild bird subtypes that have been associated with severe disease in poultry (H5 and H7 subtypes) and enter them into the database, it also implies that subtypes seen in multiple genera are more likely to infect and persist in poultry.

We found that some subtypes appear to be limited to certain bird orders or flyways, which suggest the presence of a limited degree of subtype specificity to host or geographic region, but may also reflect sampling biases within GenBank. Fifty-six percent (5/9) of H9 subtypes were only found in Charadriiformes, of which four, H9N4, H9N5, H9N6, and H9N7, were only detected in Delaware Bay shorebirds. Fifty percent (8/16) of N3 subtypes were only found in Anseriformes. Australia alone had 75% (3/4) of known H15 subtypes; the other H15 subtypes have not been observed to date. We also identified H8, H13, and H15 subtypes where four or more combinations with NA subtypes had not been observed. Noticeably, N7 lacked eight combinations with HA subtypes.

#### ii. Deficiencies and strengths of the study

A drawback of using data from GenBank for large-scale analyses is that much of the data may be biased towards the detection of HPAI subtypes. This is because of the international requirement to report all H5 and H7 viruses found in domestic and wild birds to the World Organisation for Animal Health (OIE) and to determine their pathogenicity, often by gene sequencing [22]. Thus, much AIV surveillance has been targeted on H5 and H7 subtypes and the genetic diversity uncovered was likely not characteristic of low pathogenic avian influenza subtype diversity in the landscape [23]–[25].

GenBank and the US National Center for Biotechnology Information's Influenza Virus Sequence Database represent a vast resource for researchers interested in virus populations, evolution, transmission, and movement [1], [26]–[31]. Without such repositories of AIV sequence data these types of genetic and risk analyses would not be possible. However, because they are designed as repositories of genetic information and not as repositories of surveillance information, much of the demographic and geographic data, especially regarding the host genus, is not recorded and thus is not available for analysis; nor are data about the sampling effort that generated the isolate. The absence of these data greatly diminishes the analytical, social, and economic value of the sequence data placed in these databases. In more recent years the Influenza Research Database (IRD) has rectified some of these reporting issues across multiple institutions, by incorporating standardized bird taxonomy, metadata standards, positive and negative reports, and spatial coordinates of sampling [32]. Likewise, we recommend genetic databases to at least map host species information to a standardized taxonomic database such as those maintained by BirdLife International [33].

### b. Estimating total richness for AIV subtypes

#### i. Observations and findings

The estimated subtype richness and 75% target sample size of each study vary according to factors such as location (e.g. breeding, wintering, pre-migratory gathering, or stopover sites), sampling season, size and diversity of host populations, sampling effort, sampling methodology, and laboratory diagnostics. Although we focused on studies with the largest sample sizes, differences in sampling approach or laboratory diagnostic technique between independent studies may bias comparisons of predicted subtype richness. In our interpretation of the results below we discuss those biases in specific and general terms.

Predicted subtype richness was highest in Sweden (Figure 3a). However, this did not correlate with the largest sample size required to identify 75% of the predicted virus subtypes in circulation (Figure 3b). Notably, the Swedish study only sampled Mallards, a species that the analysis of GenBank data suggests has a particularly high subtype richness. Hence, the predicted subtype richness is likely a reflection of sampling Mallards and is not related to AIV subtype diversity that may have existed among other sympatric bird species, which were present but not sampled. Moreover, within the metapopulation of Mallards that share this same flyway, AIV prevalence in Sweden was roughly 10% higher throughout the year than it was in The Netherlands [18]. This may explain why, in our study, predicted AIV subtype richness was nearly twice as high in Sweden (where the AIV prevalence was 13%) as it was across Europe (prevalence 3%). Indeed higher prevalence in a sampled population translates into more opportunities to detect subtype diversity.

Taiwan and Canada had similar predictions of subtype richness (Figure 3a) but the sample size required to achieve the 75% target for Taiwan was roughly twice that required for Canada (Figure 3b). This was likely due to the particularly low virus prevalence in Taiwan (>1%), compared to Canada (24%), such that considerably more Taiwanese birds required sampling in order to detect 75% of subtypes present. Since many different bird species/orders were sampled in Taiwan, another explanation may be that surveillance in Taiwan was not focused on the species with the greatest subtype diversity.

Interestingly, the predicted AIV subtype richness in Sweden, where the prevalence of AIV (all subtypes) was 13% in Mallards, was nearly twice the predicted richness of Canada, even though Canada had much higher AIV prevalence in “mainly Mallards” [34]. Possible explanations for the absence of a consistent, positive covariance of prevalence with subtype richness include geographic and seasonal differences, varying AIV metapopulation features such as inter-flyway connectivity, and differing laboratory methods.

We found that the sampling effort in the Northern Hemisphere (n = 224,759) was five times greater than the sampling effort in the Southern Hemisphere (n = 43,768). Despite higher sampling effort in Africa and Australia than the North American Flyway regions (Figure 2a), reported subtype richness in Africa and Australia was almost 50% less (Figure 2b). This supports the concept that Australia has its own AIV lineages [35]. Because of the large differences in sampling effort, differences in targeted species, and possible GenBank reporting bias, it should not be suggested that sampling for global surveillance for circulating AIV subtypes should be centered in the Northern Hemisphere. Certainly neglecting the Southern Hemisphere in surveillance efforts would lead to a failure to detect any subtypes specific to that region, which may have its own unique AIV ecology.

For the Northern Hemisphere, our findings show that across seven studies the estimated sample size required to detect the 75% of subtypes in circulation ranged from 10,000 to 50,000 birds. If we consider the target sample sizes for some of the different locations represented in our data, one feasible surveillance approach could involve selecting 3–5 study locations, replicating the associated study methodology, and sampling 10,000–20,000 birds at each. Such a survey could be done over multiple years to account for annual variability in circulating subtypes and to make achieving the required sample size more manageable. Because of the lower sample sizes needed in Canada and Sweden, where AIV prevalence has been high in past surveys, these may be good locations on which to focus a Northern Hemisphere surveillance effort. In North America, others have suggested that the northern prairie pothole region is an important staging and mixing area for ducks, and thus for AIV surveillance [36]. Focused sampling to obtain the bulk of subtypes should not overlook the importance of exploratory studies to detect missing or rare subtypes in understudied regions and species.

#### ii. Factors that affect the likelihood of hosts carrying viruses or of virus detection

We recognize the need to be cautious when comparing different studies with different methodologies and populations, and to be aware of how these factors are influencing the system when we describe general patterns. Comparable detections of AIV carriage will depend on our ability to sample a representative part of the host populations, and therefore of the virus population, at a given time.

The marked seasonal variations in AIV prevalence are associated with stages in the bird host life cycle [37]. Transmission to new hosts is facilitated by breeding and the incorporation of immunologically naïve individuals into the population. Levels of population immunity will determine the seasonal AIV prevalence and subtype dynamics [38]. In the Northern Hemisphere, more virus has been detected from hatch year birds during their first southward fall migration than the spring northward migration. Virus prevalence and detection is greater at higher latitudes and decreases as the birds move into the wintering grounds [18], [39]–[41]. The congregation of birds during migration and at stopover sites increases transmission and spread. Populations of some species support sustained AIV circulation and have a major role as reservoir hosts while other species may be spillover hosts. Therefore, a study's ability to detect subtypes will depend on what host species are sampled and where and when they are sampled within this annual cycle.

The duration and intensity of virus excretion by infected birds may affect the detection of viruses. Typically, AIV infections are acute, with relatively short excretion periods (from seven days up to 20 days in experimental infections of naïve ducks [17], [42], [43]. Therefore time of sampling and type of sampling (capture method, cloacal vs. respiratory, and individual vs. environmental) during the course of infection in a single individual will affect the probability of detecting virus [44]. The quality of the samples collected and the type of sample analysis also affects virus detection and culture. Factors that influence isolation success are the composition of transport medium, sample storage temperature, and the number of freeze and thaw cycles [16]. Virus diversity data are mostly based on those isolated in embryonated chicken (*Gallus gallus domesticus*) eggs, and some AIVs may grow poorly in these eggs – for instance gull AIVs that seem to be host specific [45].

#### iii. Assumptions and opportunities

It is clear that it is not currently feasible to obtain a perfectly representative sample of host species and their AIV diversity. We set reasonable inclusion criteria for the data sets used in this analysis, and thereafter assumed that the seven case studies selected were sufficiently representative of host and virus populations and sufficiently comparable to warrant a descriptive assessment of likely drivers. Notably, the Southern Hemisphere was not represented within these seven studies.

Not surprisingly, our examination of factors that influence richness demonstrated the importance of effort alongside prevalence and study duration and interactions between the three. Unfortunately data were too limited to enable a careful examination of temporal and interactive drivers of richness (e.g. local species composition, sampled population, location, seasonality, and prevalence within taxa) but it is an area of important research.

## Conclusions and Recommendations

### a. Future AIV surveillance and studies in wild birds

Based on current data availability, sample-based accumulation curves provide an initial rationalization and optimal (cost-effective) strategy for AIV surveillance, with the intention of identifying a high proportion of the virus subtypes in circulation in a given time interval. Presently, researchers can select species, locations, and months that are going to maximize the diversity pool, but sample-based accumulation curves will allow them to (1) estimate the size of the virus subtype diversity pool once sampling is underway, and (2) then optimize their sampling strategy to maximize subtype detection while minimizing samples collected and tested.

Our GenBank results also provide perspective on geographic and host species distributions of AIV relevant to global surveillance. The global effort analysis we conducted identified significant sampling bias between the hemispheres that may partially explain the imbalance of subtypes only found in the south (n = 4) or north (n = 34). Another explanation is offered by a recent ecological niche model that showed the relative occurrence of AIV is much higher in wild bird populations in northern regions [46]. The imbalance may also be a function of host species distribution, especially Anseriformes and Charadriiformes, environmental and landscape factors, or a combination of both.

Broadly our GenBank data analysis suggests that AIV surveillance to detect the widest possible range of virus subtypes should target the orders Anseriformes and Charadriiformes, which appear to support the greatest subtype diversity. Further studies are required to determine if and how subtype diversity varies at different times of year. Our sample size estimates to detect 75% of virus subtypes in circulation are based on surveillance periods with at least five sampling periods. For some well-studied locations, with prior knowledge of spatial and temporal patterns, AIV prevalence can be used to determine sample size more precisely [7].

A more detailed and comprehensive global understanding of AIV richness drivers beyond the obvious factors of effort, AIV prevalence, and surveillance duration and location, will require researchers to provide and compile individual bird-level data [32]. Important demographic information required for meaningful eco-epidemiological analysis, such as host species, sampled location, date of sampling, age, and sex, cannot be easily obtained from published research. A specific database should be compiled to capture such detailed information and to insure comparability across studies. At a more localized scale, further data from sampling individual birds, including antibodies to AIV, and information on movement both prior to capture (e.g. through use of stable isotopes to map natal origin of bird populations [15]), and following release (e.g. through banding, color marking, or various forms of telemetry) will also be highly valuable for future modeling.

Ultimately, the selection of approaches to surveillance will depend on the objectives of the individual programs, and a combination of strategies would be required to address additional research questions. A coordinated strategy of wild bird surveillance could be employed to describe a desired proportion of AIV subtype richness in a cost effective manner. This surveillance would not detract from the importance of exploratory studies (e.g. using serology [47]), which seek to identify new pools of virus diversity that may support unique subtypes (such as the recent isolation of H14 viruses from sea-ducks in Wisconsin [48] or H15N4 in western Siberia [49]).

### b. Subtype diversity: high throughput sequencing and analysis

In the future, sample-based accumulation curves could be used to assess what areas and species to focus on (e.g. a judicious mix of Mallards and exploratory studies) in order to detect the widest diversity of AIV subtypes as possible for sequencing. Although the number of available complete AIV genome sequences is still relatively small compared to those of HA and NA subtype gene segment data, the increasing capability and capacity of high-throughput genome sequencing tools now makes possible full-genome sequencing at unprecedented speed and ever lower cost. Thus, it is now possible to focus AIV surveillance on the whole spectrum of genetic diversity rather than on HA and NA subtypes alone. Such full genome data will advance our understanding of the molecular evolution, epidemiology, pathogenicity, transmission, and antiviral resistance among AIV. Once high throughput facilities have led to a better understanding of diversity within the genome constellation, we can again use sample-based accumulation curves to iteratively improve predictions of transient AIV subtype diversity and estimate host sampling targets.

### c. Broader perspective: recommendations for general pathogens

This analysis has pioneered the application of species accumulation curves to estimating the surveillance effort required to monitor the subtype diversity and richness of AIV. A collaborative international surveillance program based on this analysis would help meet most of the animal and human health objectives of AIV surveillance world-wide and contribute to vigilance and preparedness for potential influenza A pandemics as urged by Morse *et al.* [4].

More broadly, while we have focused on analyzing subtype richness within a single virus group (avian influenza A viruses), the approach is applicable to analysis of higher-level virus taxa (e.g. species, genus, family, or order) as well as to other pathogens such as other viruses (e.g. West Nile), bacteria, or helminths. Examining sampling effort in relation to novel pathogen identification can provide estimates of necessary sample sizes to detect a target number of pathogen types. Requiring relatively minimal data, species accumulation curves can be applied to expand our knowledge of pathogen diversity.

## Supporting Information

Figure S1 **Decline in subtyped AIV sequence submissions to GenBank in both poultry and wild birds.**
(TIF)

Figure S2 **The minimum sufficient sample size for capturing 75% of AIV subtypes at a location and the total estimated richness for sites with 95% confidence intervals.** Locations with small minimum sample size targets but high total estimated richness will detect more subtype diversity per bird sampled than locations with larger minimum sample size targets but lower total estimated richness.
(TIF)

Table S1 **Northern hemisphere surveillance summary of avian influenza subtype richness studies from published literature.**
(PDF)

Table S2 **Southern hemisphere surveillance summary of avian influenza subtype richness studies from published literature.**
(PDF)

Table S3 **Data and linear mixed model summary of AIV richness and other variables.**
(PDF)

Table S4 **Wild bird AIV subtype GenBank records and AIV subtype richness by bird order.** The total subtype richness represents the number of unique subtypes within all wild bird orders and is not a summation.
(PDF)

Table S5 **Specific wild bird AIV subtype GenBank records and AIV subtype richness by bird order.** Italics indicate the subtype is shared with domestic birds.
(PDF)

Table S6 **Anseriformes AIV subtype records and richness by bird family.** Italics indicate the subtype is shared with domestic birds.
(PDF)

Table S7 **Charadriiformes AIV subtype records and richness by bird family.** Italics indicate the subtype is shared with domestic birds.
(PDF)

Table S8 **Number of subtypes found uniquely in specific flyways and/or bird orders.**
(PDF)

Table S9 **Number of subtypes found uniquely in North American flyways and/or bird orders.**
(PDF)
