# Supplementary figures and images for: Sampling Strategies and Biodiversity of Influenza A Subtypes in Wild Birds
Source: PLoS One. 2014 Mar 5;9(3):e90826. doi: 10.1371/journal.pone.0090826 (PMC3944928; doi:10.1371/journal.pone.0090826)

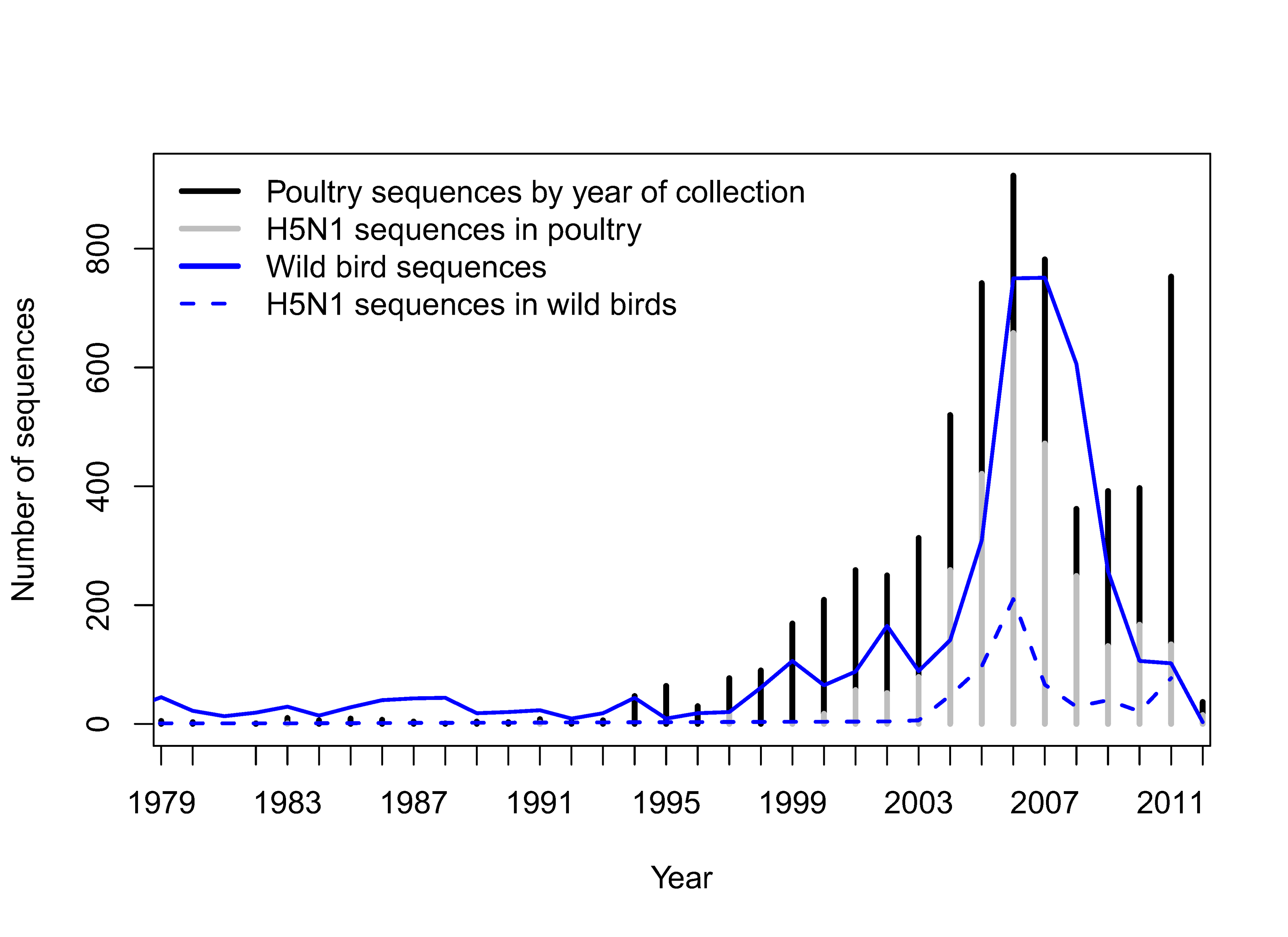

Supplement: Figure S1 — Decline in subtyped AIV sequence submissions to GenBank in both poultry and wild birds. (TIF) [file pone.0090826.s001.tif]

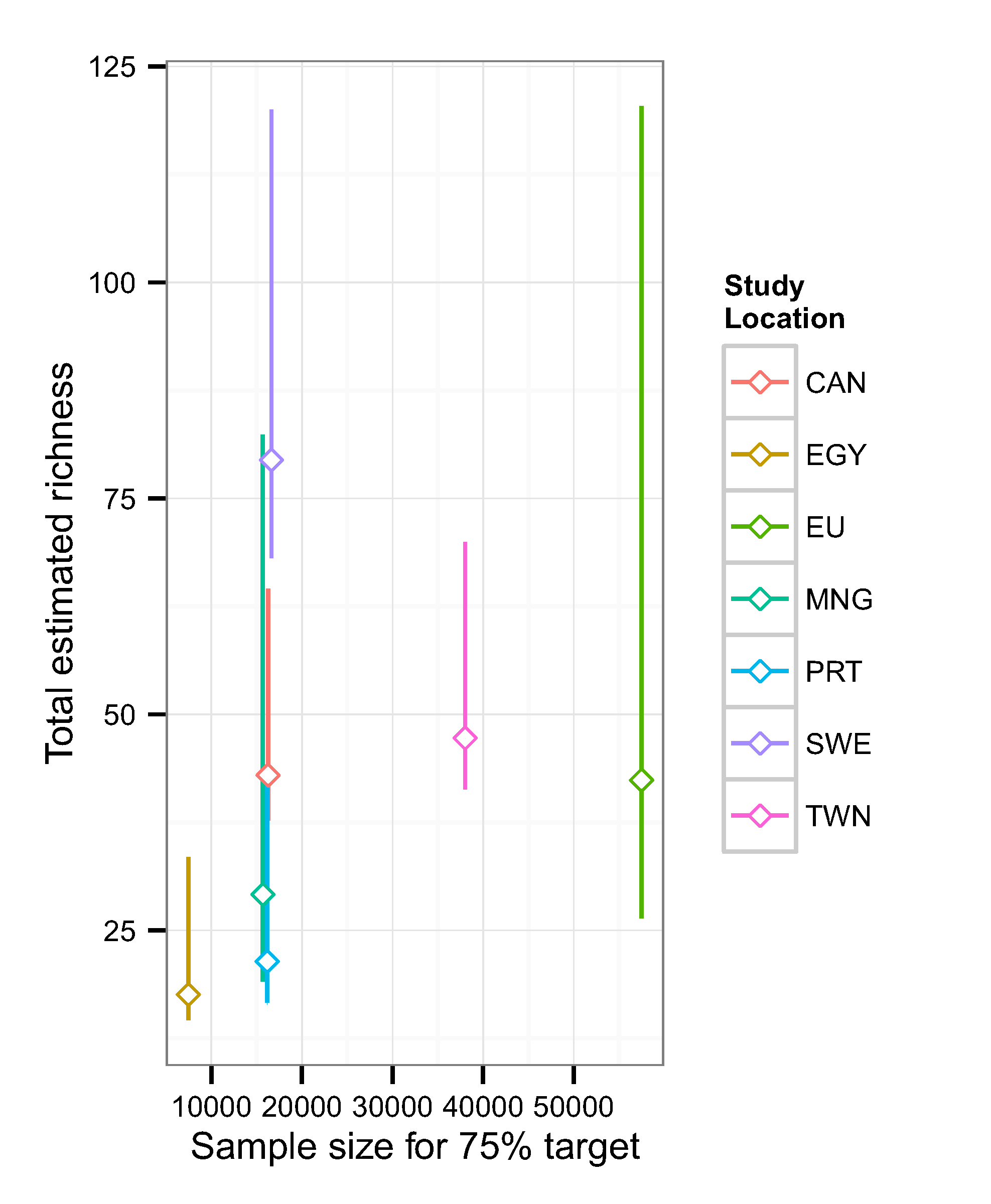

Supplement: Figure S2 — The minimum sufficient sample size for capturing 75% of AIV subtypes at a location and the total estimated richness for sites with 95% confidence intervals. Locations with small minimum sample size targets but high total estimated richness will detect more subtype diversity per bird sampled than locations with larger minimum sample size targets but lower total estimated richness. (TIF) [file pone.0090826.s002.tif]
