# Supplementary material for: Sampling Strategies and Biodiversity of Influenza A Subtypes in Wild Birds
Source: PLoS One. 2014 Mar 5;9(3):e90826. doi: 10.1371/journal.pone.0090826 (PMC3944928; doi:10.1371/journal.pone.0090826)
Supplement: Table S1 — Northern hemisphere surveillance summary of avian influenza subtype richness studies from published literature. (PDF) [file pone.0090826.s003.pdf]

Supplementary Table S1. Northern hemisphere surveillance summary of avian influenza subtype richness studies from published literature

Location (author, year)	Richness/ sample size (per 1000)	Analysis method	Sampling periods	Bird families (positive/total, % positive)
**Asia** (Marchenko et al. 2012)	6/2604 (0.64)	Cloacal swabs & intestinal samples + isolation + HI & NI test + RT-PCR + sequencing	1 (2003-2009)	Anseriformes (9/604, 1.5%)<br>Charadriiformes (1/684, 0.14%)<br>Passeriformes (2/674, 0.30%)<br>Ciconiiformes (0/300, 0%)<br>Gruiformes (0/118, 0%)<br>Podicipediformes (1/45, 2.2%)<br>Pelecaniformes (3/95, 3.2%)<br>Falconiformes (1/42, 2.4%)<br>Galliformes (0/16, 0%)<br>Columbiformes (0/18, 0%)<br>Coraciiformes (0/3, 0%)<br>Cuculiformes (0/2, 0%)<br>Piciformes (0/2, 0%)<br>Strigiformes (0/1, 0%)
**Canada** (provided by Canadian Cooperative Center for Wildlife Health 4 January 2013)	26/4484 (5.8)	Cloacal swab + real time RT-PCR + (isolation + HI & NI test + RT-PCR) +/- sequence (live & dead birds)	1 (2005)	Dabbling ducks (1484/3874, 38%)<br>Other ducks (107/454, 24%)<br>Sea ducks (2/16, 13%) – dead<br>Geese & swans (2/11, 18%) – dead<br>Gulls (6/29, 21%) – dead<br>Passerines (3/24, 13%) – dead<br>Birds of prey (1/17, 6%) – dead<br>Seabirds (0/7) – dead<br>Other (5/50, 10%) – dead
Alberta, **Canada** (Hinshaw et al. 1985)	44/9195 (4.8)	Cloacal? + isolation + HI & NI test	8 (1976, 1977, 1978, 1979, 1980, 1981, 1982, 1983)	Anatidae majority (2275/9195, 24%)
*Alberta, **Canada** (Sharp et al. 1997)	58/12321 (4.7)	Cloacal swab + isolation + HI & NI test + RT-PCR + sequence	1 (1976–1990)	Anatidae (2839/12321, 23%)
*Alberta, **Canada** (Krauss et al. 2004)	63/13466 (4.7)	Cloacal swab + isolation + HI & NI test + RT-PCR + sequence	1 (1976–2001)	Anatidae (2989/13466, 22.2%)
*Alberta, **Canada** (Krauss et al. 2012)	12/1038 (12)	Respiratory swab + isolation + HI & NI test + RT-PCR + sequence	4 (2007, 2008, 2009, 2010)	Anatidae (141/1038, 14%)
Alberta, **Canada** (Krauss et al. 2012)	25/1240 (20)	Cloacal swab + isolation + HI & NI test + RT-PCR + sequence	4 (2007, 2008, 2009, 2010)	Anatidae (153/1240, 12%)

Newfoundland, **Canada** (Wille et al. 2011)	1/38 (26)	Cloacal swab + RT-PCR + isolation + sequence	1 (2008–2009)	Laridae (2/38, 5.3%)
**China** (Zeng 2008)	12/158 (76)	Cloacal/tracheal + HI test + RT-PCR	1(2005–2006)	Anatidae (20/144, 15%)<br>Ardeidae (0/2, 0%)<br>Accipitridae (0/4, 0%)<br>Charadriidae (0/1, 0%)<br>Corvidae (0/1, 0%)<br>Falconidae (0/2, 0%)<br>Gruidae (0/2, 0%)<br>Rallidae (0/1, 0%)<br>Phasianidae (0/1, 0%)<br>Upupidae (0/1, 0%)
**Egypt** (Soliman et al. 2012)	17/6070 (2.8)	Cloacal swab + RT-PCR + isolation + heamagglutination test + sequencing	5 (2003, 2004, 2005, 2006, 2007)	Anatidae majority (9.4%)
**Europe** (Munster unpub.) used in Munster et al. 2007	29/24428 (1.2)	Cloacal swab + real-time RT-PCR + isolation + HI & NI test	8 (1998, 1999, 2000, 2001, 2002, 2003, 2004, 2005)	Total (612/24516, 2.5%)<br>*Anas platyrhynchos* (325/4398, 7.4%)
**France** (Lebarbenchon et al. 2007)	1/1044 (0.96)	Cloacal swabs & fecal + RT-PCR + isolation + HI & NI test	1 (2006)	Charadriiformes (2/102, 1.9%)<br>Accipitriformes (0/3, 0%)<br>Caprimulgiformes (0/2, 0%)<br>Ciconiiformes (0/175, 0%)<br>Columbiformes (0/3, 0%)<br>Coraciiformes (0/19, 0%)<br>Passeriformes (0/621, 0%)<br>Phoenicopteriformes (0/113, 0%)<br>Piciformes (0/3, 0%)<br>Strigiformes (0/3, 0%)
**Germany** (Hlinak et al. 2006)	2/630(3.2)	Tracheal & cloacal swab + isolation + HI & NI test	2 (2001, 2002)	Anatidae (4/50, 8%)<br>Charadriformes (0/494, 0%)<br>Gruiformes (0/4, 0%)<br>Passeriformes (0/82, 0%)
**Germany** (Süss et al. 1994) used in Sinnecker et al. 1983	28/19191 (1.5)	Tracheal or cloacal swab + isolation + HI & NI test	1(1977-1989)	Total wild/feral only (325/19191, 1.7%)
**Guatemala** (González-Reiche et al. 2012)	6/256 (23)	Tracheal & cloacal swabs + RT-PCR + isolation + sequencing	4 (2006–2007, 2007–2008, 2008–2009, 2009–2010)	Anatidae (28/234, 12%)<br>Picidae (1/21, 4.8%)<br>Tyrannidae (1/1, 100%)
**Iran** (Fereidouni et al. 2010)	13/1146 (11)	Oropharyngeal/cloacal + isolation + RT-PCR + HI & NI test +	3 (2003/2004, 2005, 2007)	Podicipedidae (0/19, 0%)<br>Phalacrocoracidae (0/14, 0%)

		sequencing/microarray		Ardeidae (0/22, 0%)<br>Phoenicopteridae (0/12, 0%)<br>Anatidae (31/745, 4.2%)<br>Rallidae (4/234, 1.8%)<br>Recurvirostridae (0/6, 0%)<br>Charadriidae (0/17, 0%)<br>Scolopacidae (0/51, 0%)<br>Laridae (0/25, 0%)<br>Sternidae (0/1, 0%)
**Italy** (De Marco et al. 2005)	3/638 (4.7)	Cloacal swab + isolation + HI & NI test	6 (Jan-Mar 1998, Dec 1998-Jan 1999, Jun 1999, Dec 1999, Feb 2000, Jun 2000)	Anatidae (5/326, 1.5%)<br>Gruiformes (0/162, 0%)<br>Laridae (0/133, 0%)
**Italy** (Terregino et al. 2007) used in Cattoli et al. 2007	15/4083 (3.7)	Cloacal swab + RT-PCR + isolation + sequencing	1 (2004–2006)	Total (327/4083, 8.0%)<br>Anatidae majority
**Japan** (Fujimoto et al. 2010)	12/4335 (2.8)	Fecal sample + isolation + HI & NI test + RT-PCR + sequencing	8 (2001, 2002, 2003, 2004, 2005, 2006, 2007, 2008)	Anatidae (41/4309, 0.95%)<br>Laridae (0/26, 0%)
**Mongolia** (provided by M. Gilbert 10 February 2013) used in Gilbert et al. 2012	23/5831 (3.9)	Fecal sample + isolation + HI & NI test + RT-PCR + sequencing	15 (Jul 2009, Aug 2009, Sep 2009, May 2010, Jun 2010, Jul 2010, Sep 2010, May 2011, Jun 2011, Jul 2011, Aug 2011, Sep 2011, Oct 2011, Sep 2012, Oct 2012)	Anatidae (80/5731, 1.4%)<br>Laridae (0/100, 0%)
**Portugal** (Henriques et al. 2011)	20/5691 (3.5)	Cloacal/oropharyngeal+ mRT-PCR + isolation + sequencing	5 (2005, 2006, 2007, 2008, 2009)	Total (93/5691, 1.63%)
**Portugal** (Tolf et al. 2012)	8/1632 (4.9)	Cloacal + RT-PCR + isolation + HI & NI test + sequencing	1 (2008-2009)	Total (72/1653, 4.4%)<br>*Anas platyrhynchos* (69/1542, 4.5%)<br>*Anas crecca* (2/56, 3.6%)<br>*Anas clypeata* (0/30, 0%)<br>*Anas penelope* (0/12, 0%)<br>*Anas strepera* (1/8, 12.5%)<br>*Aythya fuligula* (0/5, 0%)

**Russia** (Sivay et al. 2012)	8/5678 (1.4)	Cloacal and fecal (individual and pooled) + isolation + HI & NI test + PCR + sequencing	1 (2008)	Anatidae (30/2017, 1.5%)<br>Araeidae (1/81, 1.2%)<br>Laridae (10/1553, 0.64%)<br>Rallidae (1/164, 0.61%)<br>Podicipediformes (0/64, 0%)<br>Scolopacidae (0/334, 0%)<br>Alcidae (0/19, 0%)<br>Charadriidae (0/190, 0%)<br>Recurvirostridae (0/1, 0%)<br>Stercorariidae (0/1, 0%)<br>Columbiformes (0/25, 0%)<br>Passeriformes (0/989, 0%)<br>Galliformes (0/3, 0%)<br>Strigiformes (0/5, 0%)<br>Accipitriformes (0/8, 0%)<br>Coraciiformes (0/26, 0%)<br>Apodiformes (0/1, 0%)<br>Piciformes (0/3, 0%)<br>Guidae (0/1, 0%)<br>Gaviiformes (0/1, 0%)<br>Pelecaniformes (0/187, 0%)<br>Procellariiformes (0/5, 0%)
**South Korea** (Kang et al. 2010)	38/28214 (1.3)	Fecal + isolation + HA assay + RT-PCR	1 (2003-2008)	Anseriformes (225/28214, 0.8%)
**Spain**, Castilla-La Mancha (Pérez-Ramírez et al. 2010)	4/1435 (2.8)	Cloacal/fecal sample + RT-PCR + isolation + sequence	1 (2005-2007)	Total (37/1435, 2.6%)<br>Anseriformes (29/628, 4.6%)<br>Charadriiformes (0/217, 0%)<br>Gruiformes (3/180, 1.7%)<br>Pelecaniformes (0/29, 0%)<br>Columbiformes (0/31, 0%)<br>Ciconiformes (3/308, 1%)<br>Passeriformes (0/24, 0%)<br>Phoenicopteriformes (2/7, 28.6%)<br>Other (0/11, 0%)
**Spain**, Catalonia (Busquets et al. 2010)	13/1347 (9.5)	Tracheal/fecal sample + RT-PCR + isolation + sequencing for HA & NA subtypes	1 (2006-2009)	Alcedinidae (0/1, 0%)<br>Anatidae (54/686, 7.9%)<br>Ardeidae (0/12, 0%)<br>Columbidae(0/9, 0%)<br>Fringillidae (0/1, 0%)

				Laridae (2/256, 0.8%)<br>Phalacrocoracidae (0/6, 0%)<br>Phasianidae (0/1, 0%)<br>Phoenicopteridae (4/154, 2.5%)<br>Porphyridae (0/12, 0%)<br>Procellariidae (0/9, 0%)<br>Rallidae (1/80, 1.3%)<br>Recurvirostridae (0/2, 0%)<br>Scolopacidae (0/4, 0%)<br>Stercorariidae (0/1, 0%)<br>Sternidae (0/2, 0%)
**Sweden** (Ottenby Bird Observatory, Latorre-Margalef et al. 2014 used also in Latorre-Margalef et al. 2013)	74/18645 (4.0)	Cloacal swab + real time RT-PCR + isolation + HI test & NA sequencing	8 (2002-2009)	*Anas platyrhynchos* (2463/18645, 13.2%)
**Switzerland** (Baumer et al. 2010)	13/2106 (6.2)	Combined pharyngeal & cloacal sample + isolation + RT-PCR + sequencing for HA & NA subtypes	1 (2006-2009)	Anseriformes (83/1697, 4.8%)<br>Gruiformes (1/179, 0.55%)<br>Charadriiformes (0/37, 0%)<br>Pelecaniformes (0/58, 0%)<br>Podicipediformes (0/34, 0%)<br>Hirundiniformes (0/67, 0%)<br>Turdiformes (0/9, 0%)<br>Sylviiformes (0/20, 0%)<br>Ardeiformes (0/2, 0%)<br>Phasianiformes (0/1, 0%)<br>Corviformes (0/1, 0%)<br>Scolopaciformes (0/2, 0%)<br>Muscicapiformes (0/1, 0%)
**Taiwan** (provided by Meng Chu 12 November 2012) used in Cheng et al. 2010	46/44786 (1.0)	Fecal sample + isolation + HI & NI test + RT-PCR + sequencing	10 (1998, 1999, 2000, 2001, 2002, 2003, 2004, 2005, 2006, 2007, 2008, 2009, 2010, 2011)	Anatidae (229/20812, 1.1%)<br>Shorebirds (3/6435, 0.05%)<br>Laridae (2/617, 0.32%)<br>Ardeidae (2/825, 0.24%)<br>Other birds (1/598, 0.17%)
**Ukraine** (Kulak et al. 2010)	7/606 (12)	Cloacal swab + isolation + HI assay + RT-PCR	5 (Nov 2006, Mar 2007, Sep 2007, Mar 2008, Sep 2008)	Anatidae (20/514, 3.9%)<br>Rallidae (0/18, 0%)<br>Columbidae (0/45, 0%)<br>Phasiandae (0/29, 0%)
Alaska, **USA** (Ito et al. 1995)	8/3120 (2.6)	Fecal + isolation + HI & NI test + RT-	4 (1991, 1992,	Anatidae (108/3120, 3.4%)

		PCR	1993, 1994)	
Alaska, **USA** – St. Laurence Island (Ramey, Pearce, Ely, et al. 2010)	13/1411 (9.2)	Cloacal swab + isolation + sequence	1 (2007–2008)	Alcidae (10/-)<br>Laridae (6/-)<br>Charadriidae (1/-)<br>Anatidae (3/-)<br>Total (20/1411, 1.4%)
Alaska, **USA** – Central and coastal AK (Ramey, Pearce, Flint, et al. 2010; Koehler et al. 2008)	18/3415 (5.3)	Cloacal swab + RT-PCR + isolation + sequence	1(2006-2008)	Anatidae (78/3415, 2.3%)<br>*Anas acuta*
Alaska, **USA** –Aleutians (Ramey et al. 2011)	14/1226 (11)	Cloacal swab + RT-PCR + isolation + sequence	1(2006-2008)	Anatidae (78/1226, 2.5%)<br>*Polysticta stelleri*
California, **USA** (Siembieda et al. 2010)	28/4421 (6.3)	Cloacal /oropharyngeal swab + real-time RT-PCR + isolation	3 (2005–2006, 2006–2007, 2007–2008)	Anseriformes (69/4618, 1.5%)<br>Ciconiiformes (7/763, 0.9%)<br>Passeriformes (5/1309, 0.4%)<br>Galliformes (2/652, 0.3%)
Louisiana, **USA** (Stallknecht et al. 1990)	12/1389 (8.6)	Cloacal & tracheal swab + isolation + heamagglutination test + HI & NI test	2 (1986, 1987, Sep–Jan months)	Anatidae (28/1389, 2.0%)
Minnesota, **USA** (Wilcox et al. 2011)	22/4893 (4.5)	Cloacal swabs + HI & NI test	2 (2007, 2008)	Anatidae (660/4893, 13%)
Minnesota, **USA** (Lebarbenchon et al. 2010)	1/81 (12)	Cloacal swabs + isolation + HI & NI test + RT-PCR	1 (2007–2008)	Pelecaniformes (2/81, 2.4%)
New York, **USA** (Hinshaw et al. 1985)	23/1560 (15)	Cloacal? + isolation + HI & NI test	6 (1978, 1979, 1980, 1981, 1982, 1983)	Anatidae majority (155/1560, 9.9%)
New Jersey & Delaware, **USA** (Krauss et al. 2004)	71/4266 (17)	Mostly fecal (some cloacal) + pooled + + isolation + HI & NI test + RT-PCR + sequence	1 (1985-2000)	Charadriiformes (606/4266, 14.2%)<br>*Larus atricilla* and *L. argentatus* majority
New Jersey & Delaware, **USA** + some Argentina/Chile/Bermuda & (Hanson et al. 2008)	40/9402 (4.3)	Cloacal swabs & fecal + isolation + HI & NI test	1 (1999-2005)	Haemotopodidae (0/84, 0%)<br>Recurvirostridae (0/6, 0%)<br>Charadriidae (0/45, 0%)<br>Scolopacidae (291/8278, 0.35%)<br>Laridae (1/989, 0.1%)
Ohio, **USA** (Slemons et al. 1991)	20/928 (22)	Cloacal swab + isolation + heamagglutination test + agar gel diffusion + as per Beard 1980	3 (1986, 1987, 1988, fall seasons)	Anatidae (55/928 ,5.9%)
Pennsylvania, **USA** (Alfonso, Cowen, and Van Campen	6/330 (18)	Cloacal swab + isolation + heamagglutination test + HI & NI test	2 (1990, 1991)	*Anas americana* (2/19, 11%)<br>*Anas platyrhynchos* (25/240, 10%)

1995)				Others (0/71, 0%)
Texas, **USA** (Hanson et al. 2005)	7/258 (27)	Cloacal + isolation + HI & NI test	2 (2001, 2002)	Anatidae (22/258, 8.5%)

* Removed from global study to avoid duplication of result reporting

References

Alfonso, Claudia P, Barrett S Cowen, and Hana Van Campen. 1995. "Influenza A Viruses Isolated from Waterfowl in Two Wildlife Management Areas of Pennsylvania." *Journal of Wildlife Diseases* 31 (2): 1979–185.

Baumer, Anette, Julia Feldmann, Sandra Renzullo, Matthias Müller, Barbara Thür, A Hofmann, Barbara Thu, and Matthias Mu. 2010. "Epidemiology of Avian Influenza Virus in Wild Birds in Switzerland Between 2006 and 2009." *Avian Diseases* 54 (2): 875–884. doi:10.1637/9119-110209-Reg.1.

Busquets, Núria, Anna Alba, Sebastián Napp, Azucena Sánchez, Erika Serrano, Raquel Rivas, José I Núñez, and Natàlia Majó. 2010. "Influenza A Virus Subtypes in Wild Birds in North-Eastern Spain (Catalonia)." *Virus Research* 149 (1) (April): 10–8. doi:10.1016/j.virusres.2009.12.005. http://www.ncbi.nlm.nih.gov/pubmed/20045439.

Cattoli, Giovanni, Calogero Terregino, Vittorio Guberti, Roberta De Nardi, Alessandra Drago, Annalisa Salviato, Sonia Fassina, et al. 2007. "Influenza Virus Surveillance in Wild Birds in Italy: Results of Laboratory Investigations in 2003 – 2005." *Avian Diseases* 51 (s1): 414–416. doi:10.1637/7562-033106R.1.

Cheng, M C, M S Lee, Y H Ho, W L Chyi, and C H Wang. 2010. "Avian Influenza Monitoring in Migrating Birds in Taiwan During 1998–2007." *Avian Diseases* 54 (1): 109–114. doi:10.1637/8960-061709-Reg.1.

De Marco, Maria Alessandra, Emanuela Foni, Laura Campitelli, Mauro Delogu, Elisabetta Raffini, Chiara Chiapponi, Giuseppe Barigazzi, Paolo Cordioli, Livia Di Trani, and Isabella Donatelli. 2005. "Influenza Virus Circulation in Wild Aquatic Birds in Italy During H5N2 and H7N1 Poultry Epidemic Periods (1998 to 2000)." *Avian Pathology* 34 (6) (December): 480–5. doi:10.1080/03079450500368185. http://www.ncbi.nlm.nih.gov/pubmed/16537162.

Fereidouni, Sasan R, Ortrud Werner, Elke Starick, Martin Beer, Timm C Harder, Mehdi Aghakhan, Hossein Modirrousta, et al. 2010. "Avian Influenza Virus Monitoring in Wintering Waterbirds in Iran, 2003-2007." *Virology Journal* 7 (January): 43. doi:10.1186/1743-422X-7-43. http://www.pubmedcentral.nih.gov/articlerender.fcgi?artid=2837633&tool=pmcentrez&rendertype=abstract.

Fujimoto, Yoshikazu, Hiroshi Ito, Sakar Shivakoti, Jyunya Nakamori, Ryota Tsunekuni, Koichi Otsuki, and Toshihiro Ito. 2010. "Avian Influenza Virus and Paramyxovirus Isolation from Migratory Waterfowl and Shorebirds in San-in District of Western Japan Form 2001 to 2008." *Journal of Veterinary Medical Science* 72 (7): 963–967.

Gilbert, Martin, Losolmaa Jambal, William B. Karesh, Amanda Fine, Enkhtuvshin Shiilegdamba, Purevtseren Dulam, Ruuragchaa Sodnomdarjaa, et al. 2012. "Highly Pathogenic Avianinfluenza Virus Among Wild Birds in Mongolia." Ed. Gavin J. D. Smith. *PLoS ONE* 7 (9) (September 11): e44097. doi:10.1371/journal.pone.0044097. http://dx.plos.org/10.1371/journal.pone.0044097.

González-Reiche, Ana S, María E Morales-Betoulle, Danilo Alvarez, Jean-Luc Betoulle, Maria L Müller, Silvia M Sosa, and Daniel R Perez. 2012. "Influenza a Viruses from Wild Birds in Guatemala Belong to the North American Lineage." *PloS One* 7 (3) (January): e32873. doi:10.1371/journal.pone.0032873.

Hanson, B A, M P Luttrell, V H Goekjian, L Niles, D E Swayne, D a Senne, and D E Stallknecht. 2008. "Is the Occurrence of Avian Influenza Virus in Charadriiformes Species and Location Dependent?" *Journal of Wildlife Diseases* 44 (2) (April): 351–61. http://www.ncbi.nlm.nih.gov/pubmed/18436667.

Hanson, B A, D E Swayne, D A Senne, D S Lobpries, J Hurst, and D E Stallknecht. 2005. "Avian Influenza Viruses and Paramyxoviruses in Wintering and Resident Ducks in Texas." *Journal of Wildlife Diseases* 41 (3): 624–628.

Henriques, Ana M, Teresa Fagulha, Sílvia C Barros, Fernanda Ramos, Tiago Luís, Miguel Fevereiro, C Barros, and Margarida Duarte. 2011. "Multiyear Surveillance of Influenza A Virus in Wild Birds in Portugal." *Avian Pathology* 50 (6): 597–602. doi:10.1080/03079457.2011.618943.

Hinshaw, V. S., J. M. Wood, R. G. Webster, R. Deibel, and B. Turner. 1985. "Circulation of Influenza Viruses and Paramyxoviruses in Waterfowl Originating from Two Different Areas of North America." *Bulletin of the World Health Organization* 63 (4): 711–9.

Hlinak, A, R U Mühle, O Werner, A Globig, E Starick, H Schirrmeier, B Hoffmann, et al. 2006. "A Virological Survey in Migrating Waders and Other Waterfowl in One of the Most Important Resting Sites of Germany." *Journal of Veterinary Medicine* 53 (3) (April): 105–10. doi:10.1111/j.1439-0450.2006.00935.x. http://www.ncbi.nlm.nih.gov/pubmed/16629720.

Ito, T, K Okazakp, Y Kawaoka, Ayato Takada, Robert G Webster, and Hiroshi Kida. 1995. "Perpetuation of Influenza A Viruses in Alaskan Waterfowl Reservoirs." *Archives of Virology* 140: 1163–1172.

Kang, H M, O M Jeong, M C Kim, J S Kwon, M R Paek, J G Choi, E K Lee, Y J Kim, J H Kwon, and Y J Lee. 2010. "Surveillance of Avian Influenza Virus in Wild Bird Fecal Samples from South Korea, 2003-2008." *Journal of Wildlife Diseases* 46 (3) (July): 878–88. http://www.ncbi.nlm.nih.gov/pubmed/20688693.

Koehler, Anson V, John M Pearce, Paul L Flint, J Christian Franson, and Hon S Ip. 2008. "Genetic Evidence of Intercontinental Movement of Avian Influenza in a Migratory Bird: The Northern Pintail (Anas Acuta)." *Molecular Ecology* 17 (21) (November): 4754–62. doi:10.1111/j.1365-294X.2008.03953.x. http://www.ncbi.nlm.nih.gov/pubmed/19140989.

Krauss, Scott, Sydney Paul Pryor, Garnet Raven, Angela Danner, Ghazi Kayali, Richard J. Webby, and Robert G. Webster. 2012. "Respiratory Tract Versus Cloacal Sampling of Migratory Ducks for Influenza A Viruses: Are Both Ends Relevant?" *Influenza and Other Respiratory Viruses* 7 (1): 93–96. doi:10.1111/j.1750-2659.2012.00359.x.

Krauss, Scott, David Walker, S Paul Pryor, Larry Niles, L I Chenghong, Virginia S Hinshaw, and Robert G Webster. 2004. "Influenza A Viruses of Migrating Wild Aquatic Birds in North America." *Vector Borne & Zoonotic Diseases* 4 (3): 177–189.

Kulak, M V, F A Ilinykh, A V Zaykovskaya, A V Epanchinzeva, I L Evstaphiev, N N Tovtunec, K A Sharshov, et al. 2010. "Surveillance and Identification of Influenza A Viruses in Wild Aquatic Birds in the Crimea, Ukraine (2006–2008)." *Avian Diseases* 54 (3): 1086–1090. doi:10.1637/9272-020510-ResNote.1.

Latorre-Margalef N, Tolf C, Avril A, Bengtsson D, Wille M, et al. (in press) 1081 viruses – long-term variation in influenza A virus prevalence and subtype diversity in migratory Mallards. Proc R Soc B Biol Sci.

Latorre-Margalef N, Grosbois V, Wahlgren J, Munster VJ, Tolf C, et al. (2013) Heterosubtypic Immunity to Influenza A Virus Infections in Mallards May Explain Existence of Multiple Virus Subtypes. PLoS Pathog 9. doi:10.1371/journal.ppat.1003443.

Lebarbenchon, Camille, Chung-ming Chang, Sylvie Van Der Werf, Jean-thierry Aubin, Yves Kayser, Manuel Ballesteros, Michel Gauthier-clerc, and Le Sambuc. 2007. "Influenza A Virus in Birds During Spring Migration in the Camargue, France." *Journal of Wildlife Diseases* 43 (4): 789–793.

Lebarbenchon, Camille, Srinand Sreevatsan, Muthannan a Ramakrishnan, Rebecca Poulson, Virginia Goekjian, Jon J Di Matteo, Benjamin Wilcox, and David E Stallknecht. 2010. "Influenza A Viruses in American White Pelican (Pelecanus Erythrorhynchos)." *Journal of Wildlife Diseases* 46 (4) (October): 1284–9. http://www.ncbi.nlm.nih.gov/pubmed/20966281.

Marchenko, Author V Y, A Y Alekseev, K A Sharshov, V N Petrov, N Y Silko, D Tserennorov, D Otgonbaatar, I A Savchenko, and A M Shestopalov. 2012. "Ecology of Influenza Virus in Wild Bird Populations in Central Asia." *Avian Diseases* 56 (1): 234–237. doi:10.1637/9834-061611-ResNote.1.

Munster, Vincent J, Chantal Baas, Pascal Lexmond, Jonas Waldenström, Anders Wallensten, Thord Fransson, Guus F Rimmelzwaan, et al. 2007. "Spatial, Temporal, and Species Variation in Prevalence of Influenza A Viruses in Wild Migratory Birds." *PLoS Pathogens* 3 (5) (May 11): e61. doi:10.1371/journal.ppat.0030061.

Pérez-Ramírez, Elisa, Xeider Gerrikagoitia, Marta Barral, and Ursula Höfle. 2010. "Detection of Low Pathogenic Avian Influenza Viruses in Wild Birds in Castilla-La Mancha (south Central Spain)." *Veterinary Microbiology* 146 (3-4) (December 15): 200–8. doi:10.1016/j.vetmic.2010.05.008. http://www.ncbi.nlm.nih.gov/pubmed/20605691.

Ramey, Andrew M, John M Pearce, Craig R Ely, Lisa M Sheffield Guy, David B Irons, Dirk V Derksen, and Hon S Ip. 2010. "Transmission and Reassortment of Avian Influenza Viruses at the Asian-North American Interface." *Virology* 406 (2) (October 25): 352–9. doi:10.1016/j.virol.2010.07.031. http://www.ncbi.nlm.nih.gov/pubmed/20709346.

Ramey, Andrew M, John M Pearce, Paul L Flint, Hon S Ip, Dirk V Derksen, J Christian Franson, Michael J Petrula, et al. 2010. "Intercontinental Reassortment and Genomic Variation of Low Pathogenic Avian Influenza Viruses Isolated from Northern Pintails (Anas Acuta) in Alaska: Examining the Evidence Through Space and Time." *Virology* 401 (2) (June): 179–189. doi:10.1016/j.virol.2010.02.006.

Ramey, Andrew M, John M Pearce, Andrew B Reeves, J Christian Franson, Margaret R Petersen, and Hon S Ip. 2011. "Evidence for Limited Exchange of Avian Influenza Viruses Between Seaducks and Dabbling Ducks at Alaska Peninsula Coastal Lagoons." *Archives of Virology* 156 (10) (October): 1813–21. doi:10.1007/s00705-011-1059-z. http://www.ncbi.nlm.nih.gov/pubmed/21766196.

Sharp, G B, Y Kawaoka, D J Jones, W J Bean, S P Pryor, V Hinshaw, and R G Webster. 1997. "Coinfection of Wild Ducks by Influenza a Viruses: Distribution Patterns and Biological Significance." *Journal of Virology* 71 (8) (August): 6128–6135.

Siembieda, Jennifer L, Christine K Johnson, Carol Cardona, and Nichole Anchell. 2010. "Influenza A Viruses in Wild Birds of the Pacific Flyway, 2005-2008." *Vector Borne & Zoonotic Diseases* 10 (8): 793–800. doi:10.1089/vbz.2009.0095.

Sinnecker, R, H Sinnecker, E Zilske, and D Köhler. 1983. "Surveillance of Pelagic Birds for Influenza A Viruses." *Acta Virology* 27: 75–79.

Sivay, Mariya V, Sofya G Sayfutdinova, Kirill A Sharshov, Aleksander Y Alekseev, Aleksander K Yurlov, Jonathan Runstadler, Aleksander M Shestopalov, et al. 2012. "Surveillance of Influenza A Virus in Wild Birds in the Asian Portion of Russia in 2008." *Avian Diseases* 56 (3): 456–463.

Slemons, R D, M C Shieldcastle, L D Heyman, K E Bednarik, and D A Senne. 1991. "Type A Influenza Viruses in Waterfowl in Ohio and Implications for Domestic Turkeys." *Avian Diseases* 35 (1): 165–173. http://www.ncbi.nlm.nih.gov/pubmed/2029250.

Soliman, Atef, Magdi Saad, Emad Elassal, Ehab Amir, Chantal Plathonoff, Verina Bahgat, Maha El-Badry, et al. 2012. "Surveillance of Avian Influenza Viruses in Migratory Birds in Egypt, 2003-09." *Journal of Wildlife Diseases* 48 (3) (July): 669–75.

Stallknecht, D E, S M Shane, P J Zwank, D a Senne, and M T Kearney. 1990. "Avian Influenza Viruses from Migratory and Resident Ducks of Coastal Louisiana." *Avian Diseases* 34 (2): 398–405. http://www.ncbi.nlm.nih.gov/pubmed/2369380.

Süss, J, J Schäfer, H Sinnecker, and Robert G Webster. 1994. "Influenza Virus Subtypes in Aquatic Birds of Eastern Germany." *Archives of Virology* 135: 101–114.

Terregino, Calogero, Roberta De Nardi, Vittorio Guberti, Mara Scremin, Elisabetta Raffini, Ana Moreno Martin, Giovanni Cattoli, Lebana Bonfanti, and Ilaria Capua. 2007. "Active Surveillance for Avian Influenza Viruses in Wild Birds and Backyard Flocks in Northern Italy During 2004 to 2006." *Avian Pathology* 36 (4) (August): 337–44. doi:10.1080/03079450701488345. http://www.ncbi.nlm.nih.gov/pubmed/17620182.

Tolf, Conny, Daniel Bengtsson, David Rodrigues, Neus Latorre-Margalef, Michelle Wille, Maria Ester Figueiredo, Monika Jankowska-Hjortaas, et al. 2012. "Birds and Viruses at a Crossroad - Surveillance of Influenza A Virus in Portuguese Waterfowl." *PloS ONE* 7 (11) (January): e49002. doi:10.1371/journal.pone.0049002. http://www.ncbi.nlm.nih.gov/pubmed/23145046.

Wilcox, Benjamin R, Gregory a Knutsen, James Berdeen, Virginia Goekjian, Rebecca Poulson, Sagar Goyal, Srinand Sreevatsan, et al. 2011. "Influenza-A Viruses in Ducks in Northwestern Minnesota: Fine Scale Spatial and Temporal Variation in Prevalence and Subtype Diversity." *PloS ONE* 6 (9) (January): e24010. doi:10.1371/journal.pone.0024010.

Wille, Michelle, Gregory J Robertson, Hugh Whitney, Davor Ojkic, and Andrew S Lang. 2011. "Reassortment of American and Eurasian Genes in an Influenza A Virus Isolated from a Great Black-backed Gull (Larus Marinus), a Species Demonstrated to Move Between These Regions." *Archives of Virology* 156 (1) (January): 107–15. doi:10.1007/s00705-010-0839-1. http://www.ncbi.nlm.nih.gov/pubmed/21053031.

Zeng, Xiangwei. 2008. "Monitoring Influenza A Virus and Newcastly Disease Virus in Migratory Waterfowls in Sanjiang Natural Reserve of Heilongjiang Province." *Acta Microbiologica Sinica* 48 (10): 1403–7.
