# Supplementary material for: Sampling Strategies and Biodiversity of Influenza A Subtypes in Wild Birds
Source: PLoS One. 2014 Mar 5;9(3):e90826. doi: 10.1371/journal.pone.0090826 (PMC3944928; doi:10.1371/journal.pone.0090826)
Supplement: Table S2 — Southern hemisphere surveillance summary of avian influenza subtype richness studies from published literature. (PDF) [file pone.0090826.s004.pdf]

Supplementary Table S2. Southern hemisphere surveillance summary of avian influenza subtype richness studies from published literature

**Location (author, year)**	**Richness/ sample size (per 1000)**	**Analysis method**	**Sampling periods**	**Bird families (positive/total, % positive)**
**Argentina** (Pereda et al. 2008)	1/2895 (0.35)	Cloacal swabs & fecal + RRT-PCR + sisolation + sequence	1 (2006–2007)	Anseriformes (- /1860)<br>Charadriiformes (-/485)<br>Ciconiiformes (-/8)<br>Columbiformes (-/14)<br>Gruiformes (-/17)<br>Passeriformes (-/45)<br>Pelecaniformes (-/97)<br>Procellariiformes (-/18)<br>Sphenisciformes (-/249)<br>Tinamiformes (-/2)<br>Total (12/2895, 0.41%)
**Argentina** (Escudero et al. 2008)	0/165 (0)	Cloacal swab + RT-PCR	1 (2004-2005)	Shorebirds (0/165, 0%)
**Australia** (Hurt et al. 2006)	2/173 (12)	Cloacal + RT-PCR + isolation + HI & NI test	1 (Nov 2004)	*Calidris acuminata* (5/97, 5.2%)<br>*Calidris ferruginea* (0/14, 0%)<br>*Calidris ruficollis* (5/14, 36%)<br>*Calidris canutus* (0/6, 0%)<br>*Limosa lapponica* (0/29, 0%)<br>*Limosa limosa* (0/13, 0%)<br>Total (10/173, 5.8%)
**Australia** (Haynes et al. 2009)	12/16303 (0.73)	Cloacal/oropharyngeal/fecal + isolation + RT-PCR + HI & NI test	2 (2005–2006, 2006–2007)	Anseriformes (6849)<br>Charadriiformes (3748)<br>Mixed Anser+Charadriiformes (5413)<br>Other birds (410)<br>Total (48/16303, 0.29%)
**Australia,** Victoria (Peroulis and O'Riley 2004)	1/605 (1.7)	Cloacal/tracheal + isolation + HI & NI test + RT-PCR	1 (2001-2002)	Anatidae (5/284, 1.8%)<br>Galliformes (0/150, 0%)<br>Columbiformes (0/133, 0%)<br>Other (0/38, 0%)
**Australia**, Western (Mackenzie et al. 1984)	6/3654 (1.6)	Cloacal + isolation + HI & NI test	1 (1977-1979)	*Anas superciliosa* (12/233, 3.6%)<br>*Tadorna tadornoides* (3/74, 4.1%)<br>*Anas gibberifrons* (3/125, 2.4%)<br>*Puffinus pacificus* (3/531, 0.56%)

				Fulica atra (1/14, 7.1%)<br>*Sterna fuscata* (1/294, 0.34%)<br>*Anous tenuirostris* (1/254, 0.39%)<br>Total (24/3654, 0.67%)
**South Africa** (Cumming et al. 2011)	2/4977 (0.40)	Cloacal and fecal + isolation + RT-PCR	1 (2007-2009)	Alaudidae (3/24, 13%)<br>Alcedinidae (1/13, 7.7%)<br>Anatidae (52/2170, 2.4%)<br>Cerylidae (1/40, 2.5%)<br>Charadriidae (12/461, 2.6%)<br>Dendrocygnidae (12/234, 5.1%)<br>Hirundinidae (1/13, 7.7%)<br>Jacanidae (15/493, 3.0%)<br>Motacillidae (2/43, 4.7%)<br>Numididae (1/23, 4.3%)<br>Passeridae (1/8, 12.5%)<br>Ploceidae (5/165, 3%)<br>Pycnonotidae (1/8, 12.5%)<br>Rallidae (7/514, 1.4%)<br>Scolopacidae (7/181, 3.9%)<br>Sylviidae (2/13, 15.4%)<br>Threskiornithidae (1/20, 5.0%)<br>Upupidae (1/3, 33%)
**Africa-Europe** (Gaidet et al. 2007)	5/5256 (0.95)	Cloacal swabs & fecal + RT-PCR + RT-qPCR	1 (2006)	African ducks (41/1455, 2.8%)<br>Eurasian ducks (93/1409, 6.6%)<br>Eurasian waders (6/688, 0.9%)<br>Rails (3/416, 0.70%)<br>Gulls (14/366, 3.8%)<br>Terns (2/151, 1.3%)
**Zambia** (Simulundu et al. 2011)	5/3094 (1.6)	Fecal + isolation + HI & NI test + sequencing	1 (2008-2009)	Total (12/3094, 0.39%)<br>Anatidae + pelicans

References

Cumming, Graeme S, Alexandre Caron, Celia Abolnik, Giovanni Cattoli, Leo W Bruinzeel, Christina E Burger, Krizia Cecchettin, et al. 2011. "The Ecology of Influenza A Viruses in Wild Birds in Southern Africa." *EcoHealth* (April 23). doi:10.1007/s10393-011-0684-z. http://www.ncbi.nlm.nih.gov/pubmed/21516374.

Escudero, G, Vincent J Munster, M Bertellotti, and P Edelaar. 2008. "Perpetuation of Avian Influenza in the Americas: Examining the Role of Shorebirds in Patagonia." *The Auk* 125 (2): 494–495. doi:10.1525/auk.2008.2408.2.

Gaidet, N, T Dodman, A Caron, G Balanca, S Desvaux, F Goutard, G Cattoli, et al. 2007. "Influenza Surveillance in Wild Birds in Eastern Europe, the Middle East, and Africa: Preliminary Results from an Ongoing FAO-led Survey." *Journal of Wildlife Diseases* 43 (3): S22–S28.

Haynes, L, E Arzey, C Bell, N Buchanan, G Burgess, V Cronan, C Dickason, et al. 2009. "Australian Surveillance for Avian Influenza Viruses in Wild Birds Between July 2005 and June 2007." *Australian Veterinary Journal* 87 (7) (July): 266–72. doi:10.1111/j.1751-0813.2009.00446.x. http://www.ncbi.nlm.nih.gov/pubmed/19573149.

Hurt, A.C., Hansbro, P.M., Selleck, P., Olsen, B., Minton, C., Hampson, A.W., Barr, I.G., 2006. Isolation of avian influenza viruses from two different transhemispheric migratory shorebird species in Australia. Arch. Virol. 151, 2301–2309.

Mackenzie, J S, E C Edwards, R M Holmes, and Hinshaw V S. 1984. "Isolation of Ortho- and Paramyxoviruses from Wild Birds in Western Australia, and the Characterization of Novel Influenza A Viruses." *Australian Journal of Exp. Biol. Med. Sci.* 62 (February): 89–99.

Pereda, Ariel J, Ma Uhart, Rcela, Alberto a Perez, María E Zaccagnini, Luciano La Sala, Julieta Decarre, et al. 2008. "Avian Influenza Virus Isolated in Wild Waterfowl in Argentina: Evidence of a Potentially Unique Phylogenetic Lineage in South America." *Virology* 378 (2) (September 1): 363–70. doi:10.1016/j.virol.2008.06.010.

Peroulis, I, and K O'Riley. 2004. "Detection of Avian Paramyxoviruses and Influenza Viruses Amongst Wild Bird Populations in Victoria." *Australian Veterinary Journal* 82 (February): 79–82.

Simulundu, Edgar, Akihiro Ishii, Manabu Igarashi, Aaron S Mweene, Yuka Suzuki, Bernard M Hang'ombe, Boniface Namangala, et al. 2011. "Characterization of Influenza A Viruses Isolated from Wild Waterfowl in Zambia." *The Journal of General Virology* 92 (Pt 6) (June): 1416–27. doi:10.1099/vir.0.030403-0. http://www.ncbi.nlm.nih.gov/pubmed/21367986.
