# Supplementary material for: Sampling Strategies and Biodiversity of Influenza A Subtypes in Wild Birds
Source: PLoS One. 2014 Mar 5;9(3):e90826. doi: 10.1371/journal.pone.0090826 (PMC3944928; doi:10.1371/journal.pone.0090826)
Supplement: Table S3 — Data and linear mixed model summary of AIV richness and other variables. (PDF) [file pone.0090826.s005.pdf]

Supplementary Table S3. Data and linear mixed model summary of AIV richness and other variables.

AIV richness and interactions: variable summary for 41 studies

Variable	Standard deviation	Mean	Median
richness	16.7	14.5	8
nbirds	5547	3793	1632
prevalence	0.0651	0.0519	0.0305
duration	2.72	2.59	2
% Anseriformes	0.396	0.615	0.650
% Charadriiformes	0.335	0.203	0.0172

AIV richness and interactions: model summary

Linear mixed model fit by restricted maximum likelihood with all variables transformed to z-scores

*Formula: richness ~ nbirds*prevalence*duration + (1|region)*

Random effects

Groups	Name	Variance	Std.Dev.s
region	(Intercept)	0.04969	0.2229
Residual		0.12858	0.3586

Number of observations: 41, groups: continent, 6

Fixed effects

	Estimate	Std. Error	t value
(Intercept)	-0.11033	0.11745	-0.939
nbirds	0.47548	0.11121	4.275
prevalence	0.31560	0.06969	4.529
duration	0.38244	0.08628	4.433
nbirds:prevalence	0.22393	0.14732	1.520
nbirds:duration	0.05323	0.10337	0.515
prevalence:duration	0.15408	0.06847	2.250
nbirds:prevalence:duration	0.02570	0.11669	0.220

ANOVA comparison of the final model to the null model

*final model: richness ~ nbirds + prevalence + duration + nbirds*prevalence*duration + (1|region)*

null model: richness ~ (1|region)

	Df	AIC	BIC	logLik	χ^2	Df	Pr(> χ^2)
**null model**	3	121	126	-57.7			
**final model**	10	50.3	67.5	-15.2	84.97	7	1.33E-15
