# Supplementary material for: Sampling Strategies and Biodiversity of Influenza A Subtypes in Wild Birds
Source: PLoS One. 2014 Mar 5;9(3):e90826. doi: 10.1371/journal.pone.0090826 (PMC3944928; doi:10.1371/journal.pone.0090826)
Supplement: Table S4 — Wild bird AIV subtype GenBank records and AIV subtype richness by bird order. The total subtype richness represents the number of unique subtypes within all wild bird orders and is not a summation. (PDF) [file pone.0090826.s006.pdf]

Supplementary Table S4. Wild bird AIV subtype GenBank records and AIV subtype richness by bird order. The total subtype richness represents the number of unique subtypes within all wild bird orders and is not a summation.

Order	GenBank records	Subtype richness
Anseriformes	3123	101
Charadriiformes	609	70
Ciconiiformes	42	2
Columbiformes	6	2
Coraciiformes	1	1
Galliformes	2	1
Gruiformes	18	7
Falconiformes	41	3
Passeriformes	95	6
Pelecaniformes	26	5
Piciformes	1	1
Podicipediformes	25	2
Procellariiformes	13	6
Strigiformes	11	1
Struthioniformes	15	7
Suliformes	5	2
Tinamiformes	1	1
Unknown order	128	27
All orders combined	4163	112
