# Supplementary material for: Sampling Strategies and Biodiversity of Influenza A Subtypes in Wild Birds
Source: PLoS One. 2014 Mar 5;9(3):e90826. doi: 10.1371/journal.pone.0090826 (PMC3944928; doi:10.1371/journal.pone.0090826)
Supplement: Table S5 — Specific wild bird AIV subtype GenBank records and AIV subtype richness by bird order. Italics indicate the subtype is shared with domestic birds. (PDF) [file pone.0090826.s007.pdf]

Supplementary Table S5. Specific wild bird AIV subtype GenBank records and AIV subtype richness by bird order. Italics indicate the subtype is shared with domestic birds.

Order	GenBank records	subtype richness
Anseriformes	3123	101 (*H1N1*, *H1N2*, *H1N3*, H1N4, H1N5, H1N6, H1N8, H1N9, H2N1, *H2N2*, *H2N3*, H2N4, H2N5, H2N6, H2N7, H2N8, *H2N9*, *H3N1*, *H3N2*, *H3N3*, H3N4, H3N5, *H3N6*, H3N7, *H3N8*, H3N9, H4N1, *H4N2*, H4N3, H4N4, H4N5, *H4N6*, H4N7, *H4N8*, *H4N9*, *H5N1*, *H5N2*, *H5N3*, H5N4, *H5N5*, H5N6, H5N7, *H5N9*, *H6N1*, *H6N2*, *H6N3*, *H6N4*, *H6N5*, *H6N6*, *H6N8*, *H6N9*, *H7N1*, *H7N2*, *H7N3*, *H7N4*, H7N5, *H7N6*, *H7N7*, *H7N8*, *H7N9*, H8N3, *H8N4*, H8N5, H8N8, *H9N1*, *H9N2*, H9N3, H10N1, H10N2 *H10N3*, H10N4, H10N5, H10N6, *H10N7*, H10N8, H10N9, *H11N1*, *H11N2*, *H11N3*, H11N5, H11N6, H11N7, H11N8, *H11N9*, H12N1, H12N2, H12N3, H12N4, *H12N5*, H12N6, H12N8, H13N2, H13N6, H13N9, H14N3, H14N5, H14N6, H15N2, H15N4, H15N9, H16N3)
Falconiformes	41	3 (*H5N1*, *H7N3*, *H7N7*)
Charadriformes	609	70 (*H1N1*, *H1N2*, H1N5, H1N6, H1N9, H2N1, *H2N3*, H2N5, H2N6, H2N7, H2N8, *H2N9*, *H3N2*, *H3N3*, *H3N6*, H3N7, *H3N8*, H4N5, *H4N6*, H4N7, *H4N8*, *H4N9*, *H5N1*, *H5N2*, *H5N3*, H5N7, *H5N8*, *H6N1*, *H6N2*, *H6N4*, *H6N6*, *H6N8*, *H7N1*, *H7N2*, *H7N3*, *H7N4*, H7N5, *H7N7*, *H7N8*, *H7N9*, *H8N4*, *H9N1*, *H9N2*, H9N4, H9N5, H9N6, *H9N7*, *H9N9*, H10N2, *H10N3*, H10N4, H10N6, *H10N7*, H10N9, *H11N2*, H11N4, H11N5, H11N6, *H11N9*, H12N4, *H12N5*, H12N9, H13N2, H13N3, H13N6, H13N8, H13N9, H14N5, H15N9, H16N3)
Ciconiiformes	42	2 (*H5N1*, *H11N9*)
Columbiformes	6	2 (*H1N2*, *H5N1*)
Coraciiformes	1	1 (*H5N1*)
Galliformes	2	1 (*H5N2*)
Gruiformes	18	7 (*H3N8*, *H4N6*, *H5N1*, *H6N2*, *H10N3*, H12N1, H16N3)
Passeriformes	95	6 (*H3N2*, *H3N8*, *H5N1*, *H7N1*, *H7N7*, *H9N2*)
Pelecaniformes	26	5 (*H3N6*, *H5N1*, *H9N1*, *H9N2*, H13N9)
Piciformes	1	1 (*H5N1*)
Podicipediformes	25	2 (*H1N2*, *H5N1*)
Procellariiformes	13	6 (*H3N8*, *H5N3*, *H6N5*, H10N8, H15N6, H15N9)
Strigiformes	11	1 (*H5N1*)
Struthioniformes	15	7 (*H5N2*, *H6N8*, *H7N1*, *H7N4*, *H7N7*, *H9N2*, H10N1)
Suliformes	5	2 (*H5N1*, *H3N8*)
Tinamiformes	1	1 (*H1N1*)
Unknown order	128	27 (*H1N1*, *H3N1*, *H3N2*, *H3N3*, H3N5, *H3N6*, *H3N8*, *H4N2*, *H4N6*, *H4N8*, *H5N1*, *H5N2*, *H5N3*, *H6N1*, *H6N2*, *H6N5*, *H6N8*, *H7N2*, *H7N7*, *H7N8*, *H7N9*, *H9N2*, H10N1, H10N4, H10N6, *H11N1*)
