# Supplementary material for: Sampling Strategies and Biodiversity of Influenza A Subtypes in Wild Birds
Source: PLoS One. 2014 Mar 5;9(3):e90826. doi: 10.1371/journal.pone.0090826 (PMC3944928; doi:10.1371/journal.pone.0090826)
Supplement: Table S6 — Anseriformes AIV subtype records and richness by bird family. Italics indicate the subtype is shared with domestic birds. (PDF) [file pone.0090826.s008.pdf]

Supplementary Table S6. Anseriformes AIV subtype records and richness by bird family. Italics indicate the subtype is shared with domestic birds.

Name	Count	Total richness (Unique)	Unique subtypes (n=33)
Anatidae (incl. stiff-tailed & whistling ducks)	226	34 (1)	**H15N2** (*T. tadornoides*, AUS, 1983)
Dabbling duck	2509	95 (23)	**H1N4** (*A. platyrhynchos*, NLD, 2006)<br>**H1N8** (*A. platyrhynchos*, NLD, 1999; *A. acuta*, USA, 2008)<br>**H2N4** (*A. platyrhynchos,* CAN, 2002)<br>**H3N4** (*A. platyrhynchos*, CAN, 1985; 2 x *A. platyrhynchos*, US, 2008)<br>**H4N1** (*A. platyrhynchos*, CAN-AB, 1977; *A. platyrhynchos*, CAN-AB, 1998; *A. acuta*, USA-AK, 2008)<br>**H4N4** (*A. platyrhynchos*, CAN-AB, 1977; 2 x *A. gracilis*, AUS, 1979; *A. platyrhynchos*, CAN-AB, 1983; *A. clypeata*, USA-MN, 2008; 2 x *A. clypeata*, USA-CA, 2008<br>**H5N4** (*A. platyrhynchos*, USA-PA, 2006; 2 x *A. discors*, GTM, 2010)<br>***H5N5*** (*A. platyrhynchos*, USA-MN, 2000)<br>**H5N6** (*A. platyrhynchos*, USA-WI, 1975; *A. platyrhynchos*, SWE, 2002)<br>***H5N9*** (*A. platyrhynchos*, USA-OH, 1987; 8 x *A. platyrhynchos*, SWE, 2002; 2 x *A. platyrhynchos*, USA-CA, 2005; *A. acuta*, USA-AK, 2006; *A acuta*, USA-CA, 2006; *A. acuta*, USA-IL, 2006; *A. platyrhynchos*, USA-MD, 2007; *A. platyrhynchos*, USA-CA, 2008; *A. acuta*, JPN, 2008)<br>***H6N3*** (*A. platyrhynchos*, CAN-AB, 1985; 3 x *A. platyrhynchos*, CAN-AB, 1990; *A. platyrhynchos*, USA-MN, 2000)<br>**H8N3** (*A. carolinensis*, USA-CA, 2007)<br>**H8N5** (*A. platyrhynchos*, JPN, 2005)<br>**H8N8** (*Anas* sp., RUS, 2009)<br>**H9N3** (*A. platyrhynchos*, IRL, 1993)<br>**H10N5** (*A. platyrhynchos*, USA-AK, 2009)<br>**H11N7** (*A. platyrhynchos*, SWE, 2002)<br>**H11N8** (*A. platyrhynchos*, SWE, 2002; *A. platyrhynchos*, NLD, 2007)<br>**H12N2** (*Anas* sp., NOR, 2006; *A. platyrhynchos*, CHE, 2006)<br>**H12N8** (*A. platyrhynchos*, NLD, 2005)<br>**H12N6** (*A. platyrhynchos*, CAN-AB, 2006)<br>**H14N3** (*A. platyrhynchos*, RUS, 1982)<br>**H15N4** (*Anas* sp., RUS, 2008)
Diving	76	16 (0)	

duck			
Seaduck	44	18 (1)	**H14N6** (2 x *C. hyemalis*, USA-WI, 2010)
Goose	83	17 (0)	
Swan	185	21 (0)	
Non-specific subtypes*	–	– (8)	***H1N3*** (2 x *A. discors*, USA-TX, 2002; 3 x Anatidae, KOR, 2006; *A. carolinensis*, USA-CA, 2006; *A. platyrhynchos*, CAN-AB, 2006; *A. acuta*, USA-AK, 2007; 2 x *A. platyrhynchos*, USA-MN, 2007; *A. acuta*, JPN, 2008; *A. clypeata*, USA-CA, 2008; *A. acuta*, USA-CA, 2010; *A. acuta*, USA-MS, 2010)<br>***H2N2*** (*A. platyrhynchos*, RUS, 1961; *A. acuta*, RUS, 1976; *A. platyrhynchos*, USA-NY, 1978; 7 x *A. platyrhynchos*, DEU, 1983; *A. platyrhynchos*, NLD, 1999; *Anser* sp., NLD, 1999; *A. platyrhynchos*, USA-MD, 2001; *A. platyrhynchos*, JPN, 2006; *A. platyrhynchos*, NLD, 2006; 2 x *A. platyrhynchos*, SWE, 2006; *A. platyrhynchos*, NLD, 2007; 2 x *A. platyrhynchos*, SWE, 2007; *A. platyrhynchos*, RUS, –)<br>**H3N9** (*A. platyrhynchos*, CAN-AB, 1993; *A. platyrhynchos*, USA-MN, 2000; *Cygnus* sp., JPN, 2001)<br>**H4N3** (*A. platyrhynchos*, CAN-AB, 1977; *A. acuta*, USA-NY, 1982; *A. crecca*, RUS, 1987; *A. discors*, CAN-AB, 1990; *A. platyrhynchos*, SWE, 2002; *A. discors*, BRB, 2004; *A. platyrhynchos*, SWE, 2005; *A. platyrhynchos*, JPN, 2006; Anatidae, MNG, 2007; *A. discors*, CAN-AB, 2007; *A. clypeata*, USA-CA, 2010; *A. platyrhynchos*, USA-CA, 2010)<br>***H6N9*** (*A. platyrhynchos*, CAN-AB, 1979; *A. acuta*, CAN-AB, 1979; *Cygnus* sp., JPN, 2001; Anatidae, CHN, 2001; *A. platyrhynchos*, NZL, 2005; *A. platyrhynchos*, USA-OH, 2009; *A. platyrhynchos*, CZE, 2010)<br>***H7N6*** (*A. clypeata*, USA-NC, 2005; 2 x *A. carolinensis*, USA-CA, 2007; Anatidae, MNG, 2007; 2 x *A. clypeata*, USA-CA, 2007; 2 x *M. americana*, CAN-NB, 2009)<br>***H11N3*** (*A. platyrhynchos*, CAN-AB, 1983; *Cygnus* sp., JPN, 1985; *A. platyrhynchos*, USA-OH, 1986; *A. platyrhynchos*, USA-OH, 1993; *A. platyrhynchos*, SWE, 2002; *A. platyrhynchos*, USA-MO, 2005; *A. platyrhynchos*, USA-WA, 2006; *A. platyrhynchos*, ESP, 207; *A. platyrhynchos*, USA-IL, 2009;<br>**H12N3** (*Anser indicus*, MNG, 2005; 2 x *T. ferruginea*, MNG, 2005; *C. cygnus*, MNG, 2005)

* identified in more than one subfamily of Anseriformes
