# Supplementary material for: Sampling Strategies and Biodiversity of Influenza A Subtypes in Wild Birds
Source: PLoS One. 2014 Mar 5;9(3):e90826. doi: 10.1371/journal.pone.0090826 (PMC3944928; doi:10.1371/journal.pone.0090826)
Supplement: Table S7 — Charadriiformes AIV subtype records and richness by bird family. Italics indicate the subtype is shared with domestic birds. (PDF) [file pone.0090826.s009.pdf]

Supplementary Table S7. Charadriiformes AIV subtype records and richness by bird family. Italics indicate the subtype is shared with domestic birds.

Name	Count	Subtype richness (Unique)	Unique subtypes (n=10)
Alicidae (Auk)	17	13 (0)	
Laridae (Gull)	149	35 (2)	**H13N3** (*L. hyperboreus* USA-AK, 2006)<br>**H13N8** (*C. ridibundus*, NDL, 2000; *C. ridibundus*, SWE, 2005; *Larus* sp., MNG, 2008)
Terns (Sternidae)	15	8 (0)	
Wader	428	57 (6)	***H5N8*** (*A. interpres*, USA-DE, 2001)<br>**H9N4** (*A. interpres*, USA-DE, 2002)<br>**H9N6** (2 x *A. interpres*, USA-DE, 1988)<br>***H9N7*** (Charadriiformes, USA-DE, 1996; *A. interpres*, USA-DE, 1999; Charadriiformes, USA-DE, 2000)<br>**H11N4** (*A. interpres,* USA-NJ, 2002, Charadriiformes, KOR, 2006)<br>**H12N9** (*C. ruficollis*, AUS, 1981, *C. ruficolis*, AUS, 1982)
Non-specific subtypes*	–	– (2)	**H9N5** (*Calidris* sp., USA-DE, 1987; *L. atricilla*, USA-DE, 1987; 4 x *A. interpres*, USA-DE, 1987; 2 x Charadriiformes, USA-DE, 2003; *Y. aalge*, USA-OR, 2005)<br>***H9N9*** (*L. atricilla*, USA-DE, 1998; *A. interpres*, USA-VI, 1998; Charadriiformes, USA-DE, 1996; *C. canutus*, UK, 2002)

* identified in more than one subfamily of Charadriiformes
