# Supplementary material for: Sampling Strategies and Biodiversity of Influenza A Subtypes in Wild Birds
Source: PLoS One. 2014 Mar 5;9(3):e90826. doi: 10.1371/journal.pone.0090826 (PMC3944928; doi:10.1371/journal.pone.0090826)
Supplement: Table S8 — Number of subtypes found uniquely in specific flyways and/or bird orders. (PDF) [file pone.0090826.s010.pdf]

Supplementary Table S8. Number of subtypes found uniquely in specific flyways and/or bird orders.

Subtypes found in:	North America only	Europe	Asia only	Australia & New Zealand	Multiple flyways	Total
Anseriformes only	9	6	5	1	12	33
Charadriiformes only	6	0	0	1	3	10
Procellariiformes only	0	0	0	1	0	1
Multiple bird orders	7	0	1	1	59	68
Total	22	6	6	4	74	112
