# Supplementary material for: Sampling Strategies and Biodiversity of Influenza A Subtypes in Wild Birds
Source: PLoS One. 2014 Mar 5;9(3):e90826. doi: 10.1371/journal.pone.0090826 (PMC3944928; doi:10.1371/journal.pone.0090826)
Supplement: Table S9 — Number of subtypes found uniquely in North American flyways and/or bird orders. (PDF) [file pone.0090826.s011.pdf]

Supplementary Table S9. Number of subtypes found uniquely in North American flyways and/or bird orders.

Subtypes found in:	Pacific Flyway	Central Flyway	Mississippi Flyway	Atlantic Flyway
Anseriformes only	2	2	2	0
Charadriiformes only	1	0	0	4
Procellariiformes only	0	0	0	0
Multiple bird orders	0	0	0	0
Total	3	2	2	4
